# Multiomics analyses decipher intricate changes in the cellular and metabolic landscape of steatotic livers upon dietary restriction and sleeve gastrectomy

**DOI:** 10.7150/ijbs.98362

**Published:** 2024-08-19

**Authors:** Shuai Chen, Qinghe Zeng, Xiurong Cai, Jiaming Xue, Guo Yin, Peng Song, Liming Tang, Christophe Klein, Frank Tacke, Adrien Guillot, Hanyang Liu

**Affiliations:** 1 Department of General Surgery, The Third Affiliated Hospital of Nanjing Medical University, Changzhou Medical Center, Nanjing Medical University, Changzhou 213000, China.; 2 Laboratoire d'Informatique Paris Descartes (LIPADE), Université Paris Cité, Paris 75014, France.; 3 Centre d'Histologie, d'Imagerie et de Cytométrie (CHIC), Centre de Recherche des Cordeliers, INSERM, Sorbonne Université, Université de Paris, Paris 75014, France.; 4 The Jackson Laboratory, Bar Harbor, ME, USA.; 5 Department of Hepatology and Gastroenterology, Charité Universitätsmedizin Berlin, Campus Virchow-Klinikum and Campus Charité Mitte, Berlin 13353, Germany.

**Keywords:** MASLD, Bariatric surgery, Immunometabolism, Liver zonation, Myeloid cells

## Abstract

Metabolic dysfunction-associated steatotic liver disease (MASLD) is a chronic, progressive liver disease that encompasses a spectrum of steatosis, steatohepatitis (or MASH), and fibrosis. Evidence suggests that dietary restriction (DR) and sleeve gastrectomy (SG) can lead to remission of hepatic steatosis and inflammation through weight loss, but it is unclear whether these procedures induce distinct metabolic or immunological changes in MASLD livers. This study aims to elucidate the intricate hepatic changes following DR, SG or sham surgery in rats fed a high-fat diet as a model of obesity-related MASLD, in comparison to a clinical cohort of patients undergoing SG. Single-cell and single-nuclei transcriptome analysis, spatial metabolomics, and immunohistochemistry revealed the liver landscape, while circulating biomarkers were measured in serum samples. Artificial intelligence (AI)-assisted image analysis characterized the spatial distribution of hepatocytes, myeloid cells and lymphocytes. In patients and experimental MASLD rats, SG improved body mass index, circulating liver injury biomarkers and triglyceride levels. Both DR and SG attenuated liver steatosis and fibrosis in rats. Metabolism-related genes (*Ppara*, *Cyp2e1* and *Cyp7a1*) were upregulated in hepatocytes upon DR and SG, while SG broadly upregulated lipid metabolism on cholangiocytes, monocytes, macrophages, and neutrophils. Furthermore, SG promoted restorative myeloid cell accumulation in the liver not only ameliorating inflammation but activating liver repair processes. Regions with potent myeloid infiltration were marked with enhanced metabolic capacities upon SG. Additionally, a disruption of periportal hepatocyte functions was observed upon DR. In conclusion, this study indicates a dynamic cellular crosstalk in steatotic livers of patients undergoing SG. Notably, PPARα- and gut-liver axis-related processes, and metabolically active myeloid cell infiltration indicate intervention-related mechanisms supporting the indication of SG for the treatment of MASLD.

## Introduction

Obesity and overweight represent significant global health challenges, affecting approximately 2 billion people worldwide 1. Obesity-related complications encompass a spectrum of metabolic syndromes, including metabolic dysfunction-associated liver diseases (MASLD), metabolic dysfunction-associated steatohepatitis (MASH) and multi-organ disorders, contributing to elevated mortality rates among affected individuals 2, 3. MASLD encompasses a range of chronic liver pathologies affecting more than a quarter of the global population, characterized predominantly by steatosis and fibrosis, with potential progression to cirrhosis and hepatocellular carcinoma and potent immune cell involvement at all disease stages 4. Despite considerable research efforts, pharmacotherapeutic targets for MASLD remain largely elusive, with only a limited number of candidates demonstrating efficacy. Only recently, resmetirom (a selective thyroid hormone receptor beta agonist) was approved by the U.S. Food and Drug Administration (FDA), as the first treatment for patients with MASH with moderate to advanced liver fibrosis, along with diet management and exercise 5.

Lifestyle modifications, such as dietary restriction (DR) and exercise, remain the mainstay of recommended therapeutic interventions in overweight and obese individuals with MASLD, with the aim of reducing body weight by at least 10% 6, 7. However, the sustainability of long-term (hypocaloric) dietary management poses several challenges, particularly for individuals with severe obesity 8. Consequently, bariatric surgery (BS) has emerged as a viable option for obese patients, particularly when conventional nutritional and behavioral therapy fails to achieve the desired therapeutic outcomes. In a randomized controlled clinical trial, BS-based interventions were superior to lifestyle intervention alone in weight loss as well as in improving histological features of MASLD such as resolution of MASH and improvement of liver fibrosis by at least 1 stage 9. Furthermore, observational studies demonstrated long-term benefits regarding cardiovascular and liver-related morbidity as well as mortality 10.

Among various surgical modalities, sleeve gastrectomy (SG) is the most frequently performed procedure, supported by robust evidence of long-term efficacy and safety 11. SG restricts nutritional intake by reducing the stomach volume and capacity, without reconstructing the gastrointestinal tract 12. Emerging studies have suggested that BS potentially mitigates lipid accumulation and halts MASLD progression 13-15. Although the primary consequence of SG is the restriction of food intake (akin to DR), SG generates remarkable influences on systemic inflammation and gut microbiota composition 16. Recent advancements in omics technologies allow for sophisticated inquiries into liver diseases from diverse angles by profiling gene expression, protein function, metabolism, and microbiome activity 17. The latest study demonstrated that SG promotes liver regenerative capacities according to results from mouse models 18. Simultaneously, our previous study demonstrated that BS exerts superior protective effects in the livers of patients with MASLD compared to DR, a finding associated with increased macrophage infiltration in BS 14. However, little has been known about metabolic and inflammatory alterations of steatotic livers after SG and DR interventions.

Here, we sought to compare the molecular and cellular consequences of DR and SG related to the amelioration of liver steatosis, using a rodent obesity-MASLD model. We implemented single-cell (sc)/single-nuclei (sn) transcriptome analysis to describe intracellular alterations and extracellular interactions, and spatial metabolomics with the assistance of artificial intelligence (AI)-based image analysis assisted in deciphering metabolite production collating immune cell zonation. This study provides novel insights into multiomics alterations in steatotic livers from patients undergoing DR and SG, which may hopefully increase our understanding of mechanistic consequences of surgery-based therapies in MASLD.

## Results

### SG induces weight loss and ameliorates non-invasive biomarkers in human patients with MASLD

**Figure 1:**
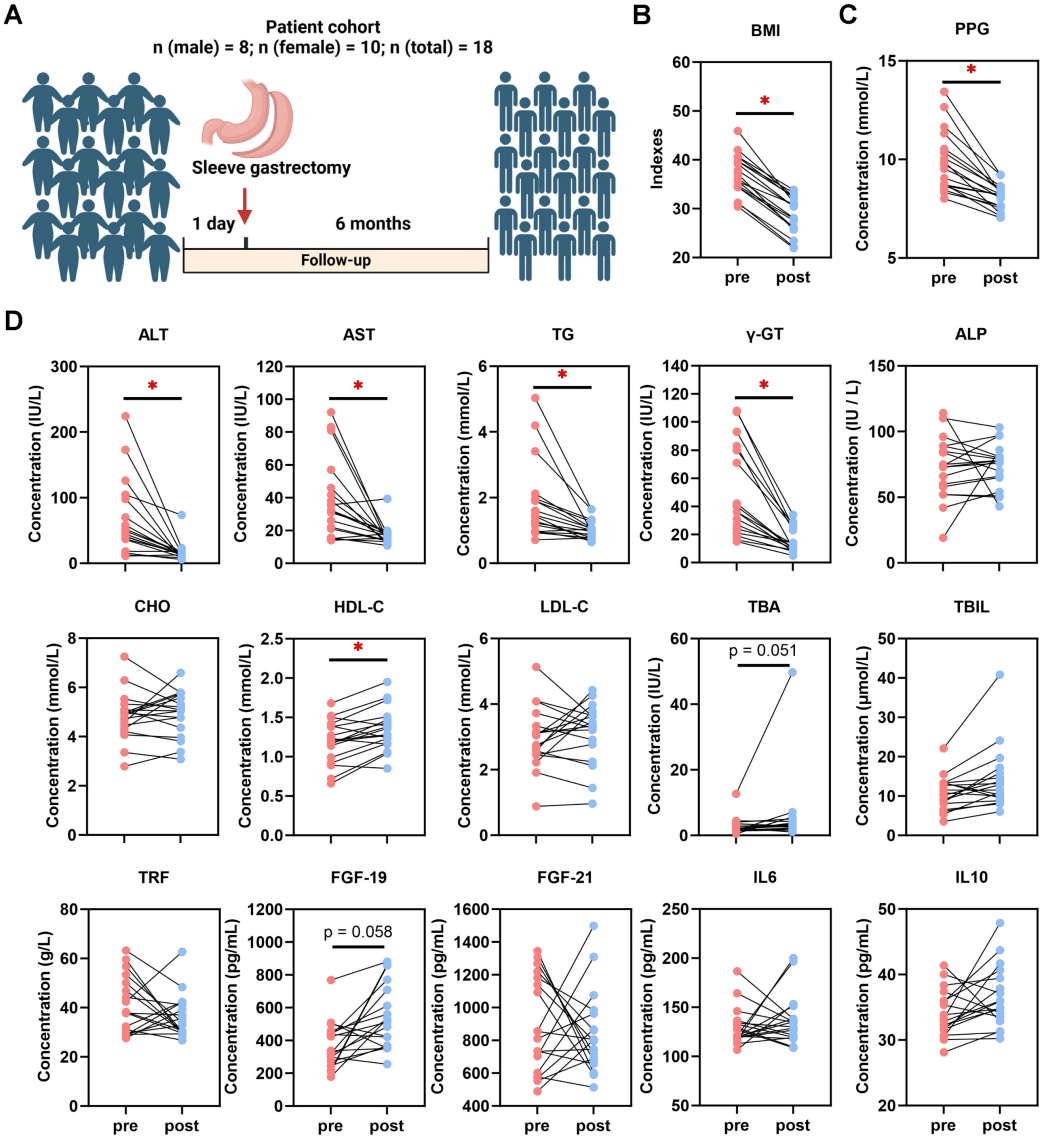
** Sleeve gastrectomy induces weight loss and MASLD amelioration in patients. (A)** Description of the patient cohort receiving SG. Comparisons of **(B)** BMI and **(C)** PPG, together with **(D)** ALT, AST, TG, γ-GT, ALP, CHO, HDL-C, LDL-C, TBA, TBIL, TRF, FGF-19, FGF-21, IL-6 and IL-10 in patients between one day pre-SG and 6 months post-SG. Abbreviations: SG: sleeve gastrectomy; BMI: body mass index; PPG: postprandial blood glucose; AST: aspartate aminotransferase; ALT: alanine aminotransferase; γ-GT: gamma-glutamyltransferase; TG, triglycerides; CHO, cholesterol; HDL-C, high-density lipoprotein cholesterol; LDL-C, low-density lipoprotein cholesterol; TBIL, total bilirubin; ALP, alkaline phosphatase; TBA, total bile acid. TRF: transferrin; IL: interleukin; FGF, fibroblast growth factor. The paired t-test was performed. '*' represents '*p* < 0.05' and statistical significance.

**Figure 2:**
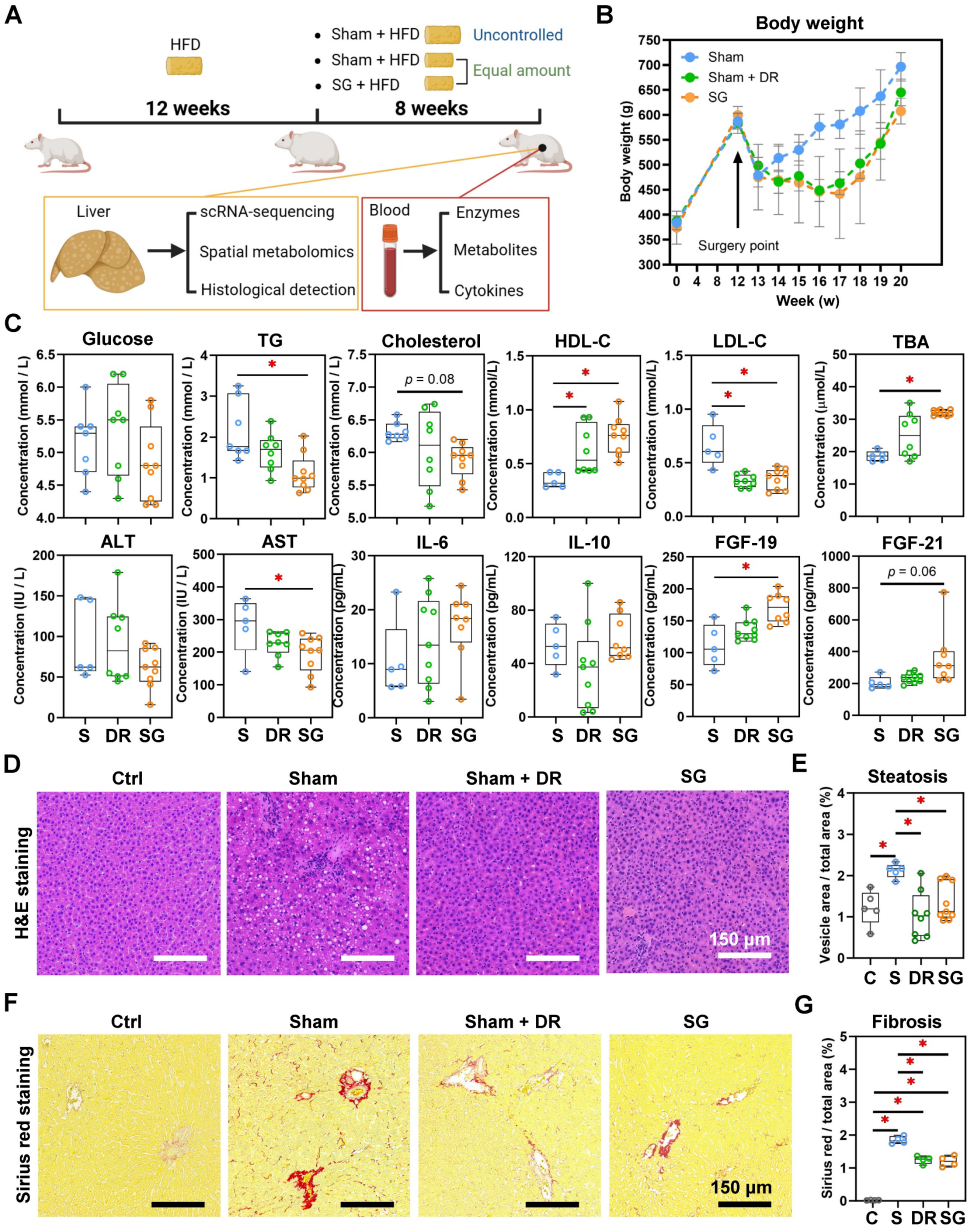
** Sleeve gastrectomy and dietary restriction induce weight loss and MASLD amelioration in rat models. (A)** The technical scheme of the rat MASLD model and surgical interventions. All animals except “Ctrl” were on HFD during this experiment. **(B)** Body weight was recorded for 20 weeks of modeling. **(C)** Serological indexes (glucose, triglycerides, cholesterol, HDL-C, LDL-C, TBA, ALT, AST, ALP, IL-6, IL-10, FGF-19 and FGF-21) were measured. **(D)** H&E-stained rat liver tissue and **(E)** quantification of steatosis (area of hepatocyte lipid vesicles) from Ctrl, Sham, Sham + DR and SG groups. **(F)** Sirius red-stained rat liver tissue and **(G)** quantification of fibrosis from Ctrl, Sham, Sham + DR and SG groups. Abbreviations: Sham/S: sham surgery; DR: dietary restriction; SG: sleeve gastrectomy; Ctrl: control fed a normal diet; HDL-C: high-density lipoprotein-cholesterol; LDL-C: low-density lipoprotein-cholesterol; TG: triglycerides; TBA: total bile acids; ALT: alanine transaminase; AST: aspartate aminotransferase; ALP: alkaline phosphatase; IL: interleukin; FGF: fibroblast growth factor. The one-way ANOVA test followed by Tukey's multiple comparisons was performed. '*' represents '*p* < 0.05' and statistical significance.

BMI and serological indexes were analyzed retrospectively in 18 patients who underwent SG (Fig. 1A). In comparison to baseline at one day pre-SG, significant declines of body mass index (BMI), glucose, postprandial blood glucose (PPG), aspartate aminotransferase (AST), alanine aminotransferase (ALT), gamma-glutamyltransferase (γ-GT), triglycerides (TG), high-density lipoprotein-cholesterol (HDL-C) and total bilirubin (TBIL) occurred at 6 months post-SG, while total bile acids (TBA) (p = 0.051) and fibroblast growth factor (FGF)-19 (*p* = 0.058) tended to increase (Fig. 1B-D, Table S2). This data hence revealed profound changes in varying processes, including inflammatory, metabolic and gut-derived endocrine signals. Intriguingly, male patients benefitted more from SG particularly on serological TG and low-density lipoprotein-cholesterol (LDL-C) levels than female patients (Table S2). Thus, to further investigate the cellular mechanisms involved in the liver response to SG and compare with DR, healthy wild-type male rats were used to recapitulate interventions on an obesity-related MASLD model (Fig. 2A, Fig. S1). All rats gained weight after 12 weeks of high-fat diet (HFD) feeding, whereas Sham + DR and SG rats had a reduced weight gain in the 8 weeks following the intervention compared to HFD alone. Moreover, DR and SG strikingly suppressed weight gain of rats from 13 to 17 weeks, while ameliorative effects compromised afterward in contrast to Sham (Fig. 2B). Serum analysis revealed that in comparison to Sham animals on HFD, Sham + DR suppressed the level of LDL but increased the level of HDL; SG significantly suppressed the levels of TG, LDL and AST, but increased the levels of HDL, TBA and FGF-19. Additionally, SG drastically suppressed cholesterol levels (*p* = 0.06) but led to increased serum FGF-21 (*p* = 0.06) (Fig. 2C). HFD exaggerated hepatic lipid accumulation in comparison to rats fed a normal diet.

Yet, both Sham + DR and SG intervention dramatically diminished hepatic lipid accumulation compared to the HFD-fed sham group (Fig. 2D and E). Simultaneously, fibrosis was increased after HFD, while it was significantly attenuated by both DR and SG (Fig. 2F and G). Results imply that both DR and SG ameliorated obesity progression, liver steatosis and fibrosis, while SG was the most efficient intervention for improving systemic levels of lipid metabolism and bile acid (BA) production.

### Sc/sn-transcriptome analysis evidences an altered hepatic cellular landscape after DR and SG

**Figure 3:**
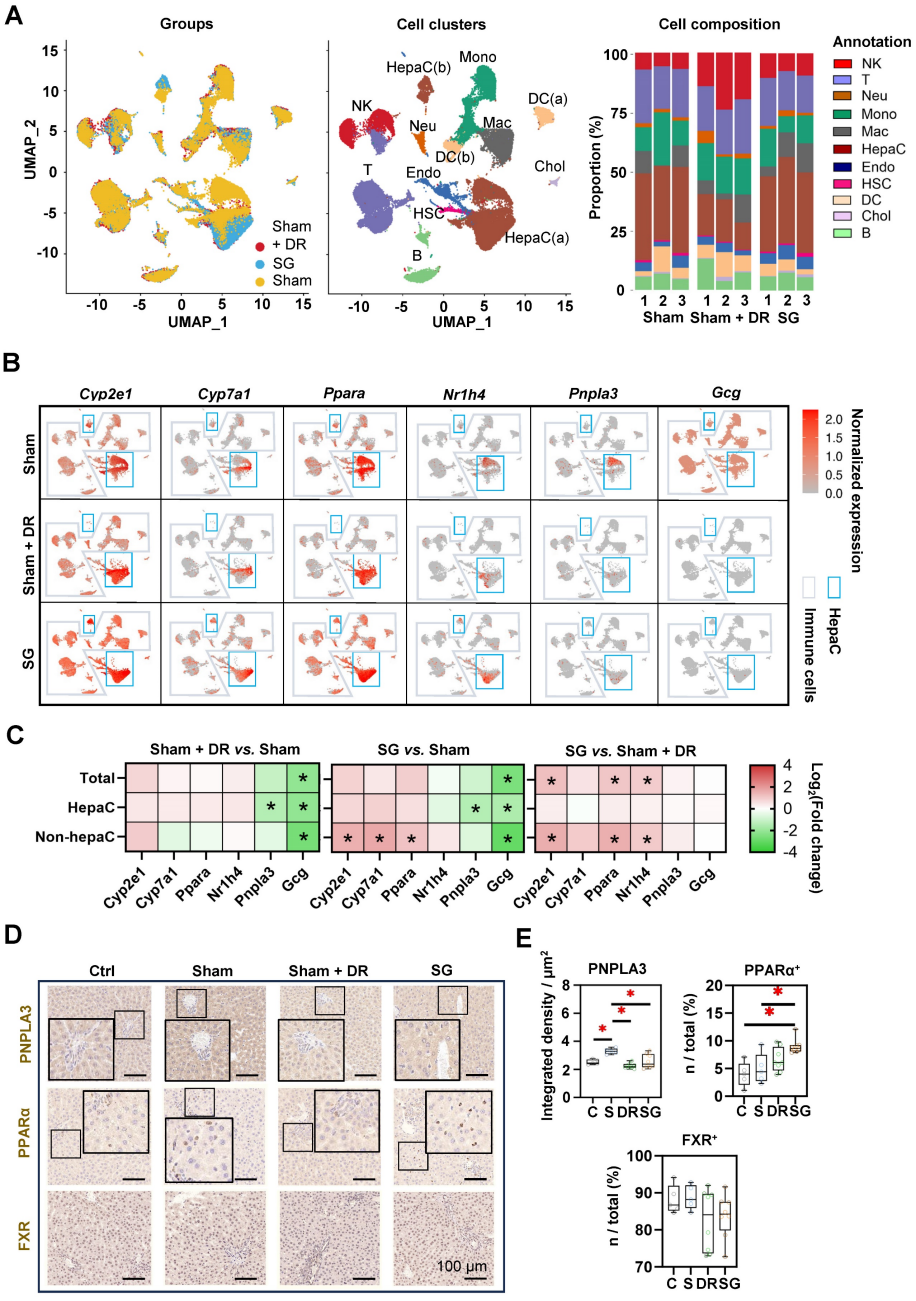
** Sleeve gastrectomy improves hepatic metabolism according to sc/sn-transcriptome analysis and in-situ exploration. (A)** UMAPs describe the distribution of B cells, cholangiocytes, DCs, endothelial cells, hepatic stellate cells, hepatocytes, macrophages, monocytes, neutrophils, T cells and NK cells. Histograms describing cell proportions. **(B)** Gene expressions of *Cyp2e1*, *Cyp7a1*, *Ppara*, *Nr1h4*, *Pnpla3* and *Gcg* were illustrated on rat liver cells upon Sham, Sham + DR and SG. **(C)** Gene expression changes of *Cyp2e1*, *Cyp7a1*, *Ppara*, *Nr1h4*, *Pnpla3* and *Gcg* were illustrated on rat liver cells upon Sham, Sham + DR and SG. Protein levels of PNPLA3, PPARα and FXR were illustrated **(D)** and quantified **(E)** on rat liver tissue of Ctrl, Sham, Sham + DR and SG groups. Abbreviations: Ctrl: control fed a normal diet; Sham: sham surgery; DR: dietary restriction; SG: sleeve gastrectomy; PPAR: peroxisome proliferator-activated receptor; PNPLA: patatin-like phospholipase domain-containing protein; FXR: farnesoid X receptor; CK: cytokeratin; IL: interleukin; DC: dendritic cells; NK: natural killer cells; B: B lymphocytes; T: T lymphocytes; Neu: neutrophils; Mac: macrophages; Mono: monocytes; Endo: endothelial cells; HSC: hepatic stellate cells; Chol: cholangiocytes; HepaC: hepatocytes. The one-way ANOVA test followed by Tukey's multiple comparisons was performed. '*' represents '*p* < 0.05' and statistical significance.

Rat liver samples [n=3 each per Sham (on HFD), Sham + DR and SG group] were analyzed using sc/snRNA-sequencing analysis, with an explicit classification of main hepatic and immune cell populations [including two populations of hepatocytes: HepaC(a) and HepaC(b), cholangiocytes, hepatic stellate cells (HSC), endothelial cells, macrophages, monocytes, neutrophils, dendritic cells (DC, two populations), B lymphocytes, T lymphocytes and natural killer (NK) cells] (Fig. 3A, Fig. S2A). Reconstituted cell composition suggested higher numbers of NK cells, B cells, neutrophils and cholangiocytes leading to a lower proportion of hepatocytes and hepatic stellate cells upon Sham + DR (*vs.* Sham). In contrast, SG intervention led to an increased proportion of macrophages, while DR led to an increased proportion of NK cells but decreased proportions of hepatocytes and HSCs (Fig. 3A, Fig. S2B). The gene expression of metabolism-related markers (*Cyp2e1*, *Cyp7a1*, *Ppara* and *Nr1h4*) and key MASLD markers (*Pnpla3* and *Gcg*) was analyzed in hepatocytes and immune cells in each group (Fig. 3B). Essentially, both Sham + DR and SG suppressed the gene expression of *Pnpla3* (mainly in hepatocytes) and *Gcg* (in total level, hepatocytes and non-hepatocytes). Nonetheless, SG increased the gene expression of *Cyp2e1*, *Ppara* and *Nr1h4* (in the non-hepatocytes) in contrast to Sham + DR (Fig. 3C). Immunohistochemistry (IHC) evidenced that patatin like phospholipase domain containing 3 (PNPLA3) protein was significantly downregulated in Sham + DR and SG groups (*vs.* Sham), while peroxisome proliferator activated receptor (PPAR)α-expressing liver cells increased in the SG group (*vs.* Sham) (Fig. 3D and E), thereby confirming the profound regulation towards beneficial metabolic pathways in hepatocytes on a protein level after SG.

**Figure 4:**
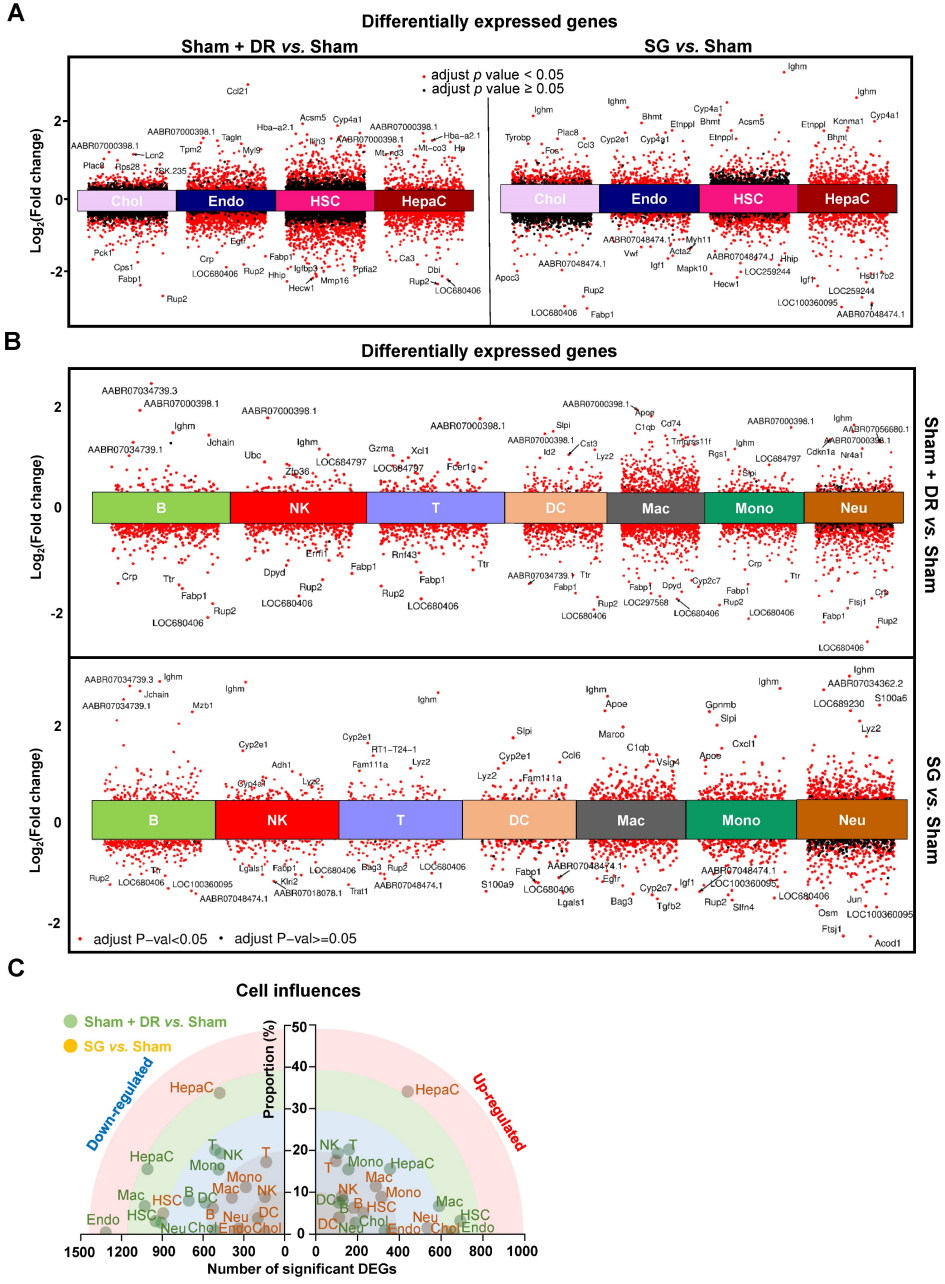
** Sc/sn-transcriptome analysis illustrates differentially expressed genes in diverse liver cells. (A & B)** Differentially expressed genes of hepatocytes, cholangiocytes, endothelial cells, hepatic stellate cells, B, T, NK, DCs, macrophages, monocytes and neutrophils (Sham + DR *vs.* Sham and SG *vs.* Sham) were illustrated in volcano plots. **(C)** Transcriptomic influences of hepatocytes, cholangiocytes, endothelial cells, hepatic stellate cells, B, T, NK, DCs, macrophages, monocytes and neutrophils in the liver were assessed with DEG numbers and cell proportion of total cells Abbreviations: Sham: sham surgery; DR: dietary restriction; SG: sleeve gastrectomy; DC: dendritic cells; NK: natural killers; B: B lymphocytes; T: T lymphocytes; Neu: neutrophils; Mac: macrophages; Mono: monocytes; Endo: endothelial cells; HSC: hepatic stellate cells; Chol: cholangiocytes; HepaC: hepatocytes; DEG: differentially expressed gene. The one-way ANOVA test followed by Tukey's multiple comparisons was performed. 'Adjust *p* < 0.05' represents statistical significance.

Furthermore, differentially expressed genes (DEGs) were screened in parenchymal (Fig. 4A) and immune cells (Fig. 4B) by comparing Sham + DR and SG to Sham. Transcriptomic influences of diverse cell types in the liver upon Sham + DR and SG were assessed based on cell proportions and the amount of significantly up-/down-regulated DEGs. Hence, the distance of dots to the origin points (value '0') are considered as the magnitude of influences (Fig. 4C). As shown in the rainbow plot, hepatocytes stand distinctly out of other cell types and as the most influential ones by SG for both gene up- and down-regulation, implying that SG impacts steatotic livers mostly through altering hepatocytic functions (Fig. 4C). Contrastingly, Sham + DR appears to result in relatively uniform effects on liver cells. In accordance with the KEGG database, enriched signaling pathways were identified based on significantly upregulated DEGs. Up- and down-regulated DEGs at the bulk levels were compared between Sham, Sham + DR and SG groups, indicating enhanced metabolic processes (SG *vs.* Sham and SG *vs.* Sham + DR) and declined metabolic processes (Sham + DR *vs.* Sham) (Fig. S3), which were in line with findings on liver samples from patients from our earlier study 14.

**Figure 5:**
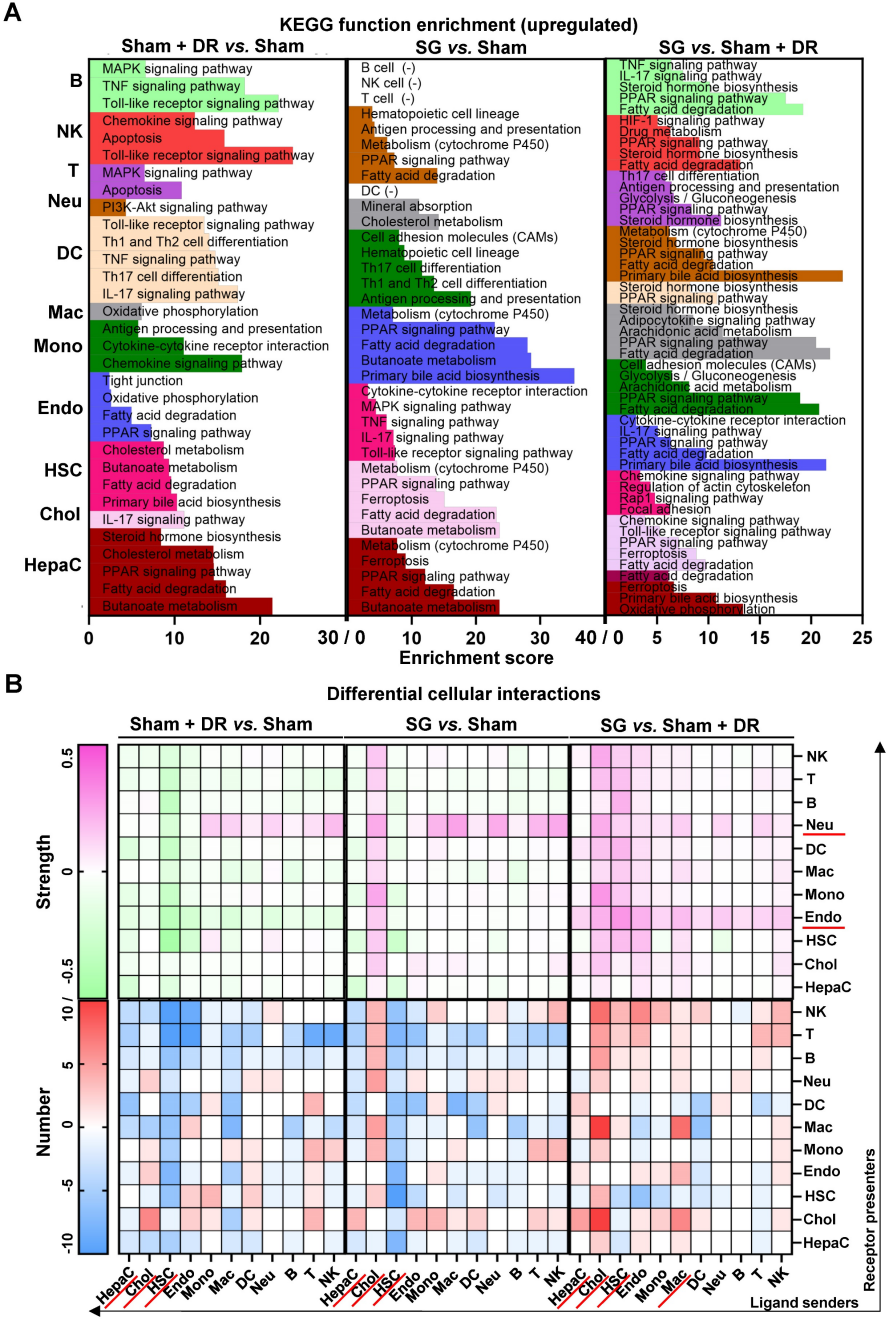
** Sc/sn-transcriptome analysis illustrates up-regulated enriched pathways and cellular interactions in diverse liver cells.** Significantly up-regulated **(A)** KEGG signaling pathways and **(B)** cellular interactions (numbers and strength) in hepatocytes, cholangiocytes, endothelial cells, hepatic stellate cells, B, T, NK, DCs, macrophages, monocytes and neutrophils were illustrated (Sham + DR *vs.* Sham and SG *vs.* Sham). Abbreviations: Sham: sham surgery; DR: dietary restriction; SG: sleeve gastrectomy; HepaC: hepatocytes; Chol: cholangiocytes; HSC: hepatic stellate cells; Endo: endothelial cells; Mono: monocytes; Mac: macrophages; DC: dendritic cells; Neu: neutrophils; NK: natural killer cells; B: B lymphocytes; T: T lymphocytes; KEGG: Kyoto encyclopedia of genes and genomes. The Fisher's exact test and the one-way ANOVA test followed by Tukey's multiple comparisons were performed. 'Adjust *p*<0.05' represents statistical significance.

Next, regulated signaling pathways were compared between the groups (Sham + DR *vs.* Sham, SG *vs.* Sham, and SG *vs.* Sham + DR) for each cell population (Fig. 5A). Fatty acid degradation-related gene expression was increased not only in hepatocytes (Sham + DR *vs.* Sham, SG *vs.* Sham, SG *vs.* Sham + DR) but also in cholangiocytes and endothelial cells (SG *vs.* Sham). Furthermore, PPAR signaling pathways, fatty acid degradation and cholesterol metabolism were more pronounced (not only in hepatocytes) by both Sham + DR and SG interventions (*vs.* Sham). Interestingly, in comparison to Sham + DR, the PPAR signaling pathway was promoted in cholangiocytes, endothelial cells and myeloid cell populations (monocytes, macrophages, DCs and neutrophils). Of note, butanoate metabolism was enhanced in hepatocytes upon both Sham + DR and SG (Fig. 5A). In addition, cellular interactions (total and ligand-receptor) were elucidated between each gradual pair of cell types among all three groups using the CellChat algorithm (Fig. S4A and B). Key signaling pathways that enriched according to total cellular interactions were described (Fig. S5A). In contrast to Sham, both Sham + DR and SG elevated ligand reception of neutrophils from immune cells, while SG elevated ligand release from cholangiocytes to other cells. Of note, in contrast to Sham + DR groups, SG promoted interaction levels of non-hepatocyte parenchymal cells (cholangiocytes, HSCs and endothelial cells) as well as immune cells (mainly for macrophages and neutrophils) (Fig. 5B). Further dissecting cholangiocyte-associated cellular interactions, significant signaling pathways from cholangiocytes to monocytes, macrophages, neutrophils, B lymphocytes, T lymphocytes and NK cells, as well as from monocytes, macrophages, DCs, hepatocytes, T lymphocytes and NK cells to cholangiocytes were illustrated (Fig. S5B and C).

Taken together, both Sham + DR and SG improve liver lipid metabolism (remarkably PPAR-associated processes) in steatotic livers. In particular, SG triggers metabolic enhancement broadly in liver cells, including hepatocytes, cholangiocytes, endothelial cells and myeloid cells. Noteworthy, cholangiocytes appear to actively interact with multiple liver cell types upon SG.

### SG promotes liver metabolic capacities through myeloid cell accumulation

**Figure 6:**
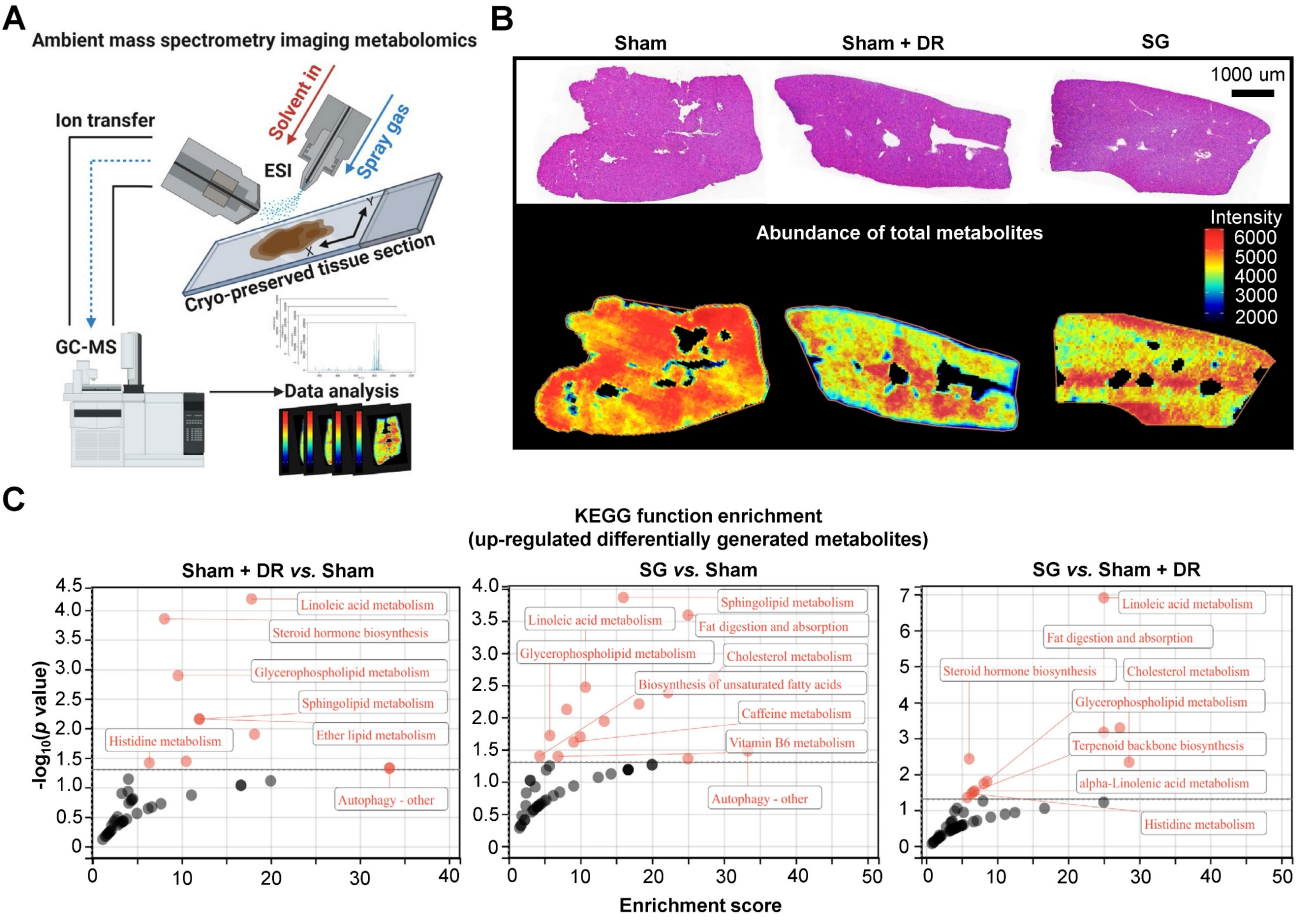
** Sleeve gastrectomy and dietary restriction enhance capacities of lipid metabolism in steatotic livers. (A)** Technical scheme of the ambient mass spectrometry imaging metabolomics method. **(B)** H&E-stained rat liver sample section was mapped with the abundance of total metabolites in Sham, Sham + DR and SG groups. **(C)** Function enrichment of upregulated differentially generated metabolites (Sham + DR *vs.* Sham, SG *vs.* Sham and SG *vs.* Sham + DR) was analyzed based on the KEGG database. Abbreviations: Sham: sham surgery; DR: dietary restriction; SG: sleeve gastrectomy; KEGG: Kyoto encyclopedia of genes and genomes. The Fisher's exact test was performed. 'Adjusted *p* < 0.05' represents statistical significance.

To explore the metabolic landscape of the liver, three representative liver samples were freshly acquired from the Sham, Sham + DR and SG groups and then analyzed using an ambient mass spectrometry imaging (MSI) metabolomics approach (Fig. 6A). The spectrum and distribution of total metabolites (with positive and negative ions) were illustrated (Fig. S6A - E). Histological features of liver samples together with spatially distributed metabolite abundance were displayed, indicating strongly reduced production of total metabolites in steatotic livers undergoing DR or SG, indicating the alleviation of global liver metabolism activities (Fig. 6B). Contrastingly, function enrichment analysis based on the total abundance of differentially upregulated metabolites elucidated that both Sham + DR and SG enhanced lipid metabolism in steatotic livers in contrast to Sham groups, potentially attenuating steatotic stress in liver. It is worth noting that SG significantly enhanced the capacities of linoleic acid metabolism, fat digestion and absorption, and cholesterol metabolism compared to Sham + DR (Fig. 6C).

**Figure 7:**
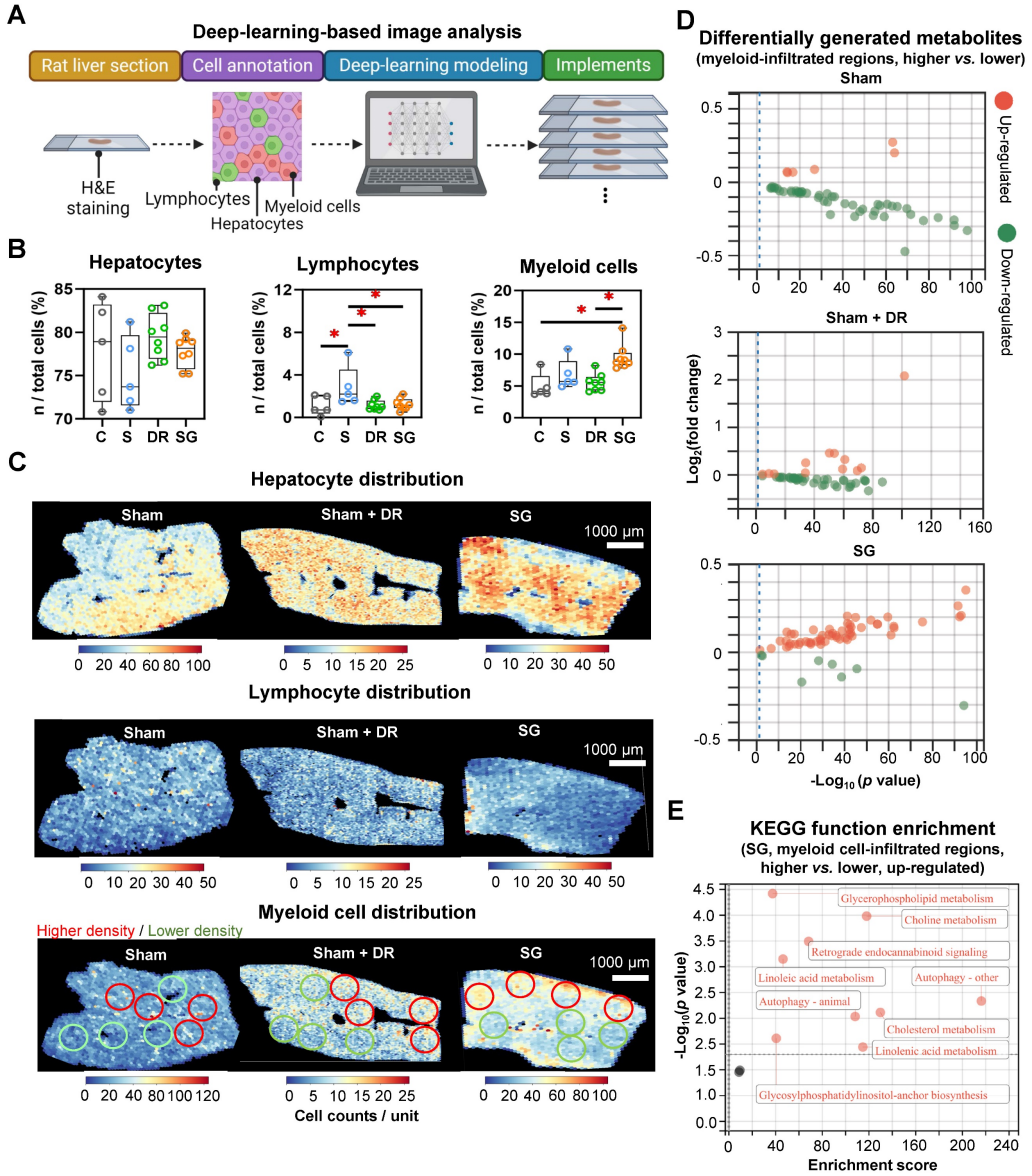
** Sleeve gastrectomy facilitates lipid metabolism associated with myeloid cell infiltration in the liver. (A)** The technical scheme of the deep-learning-based image analysis. The deep-learning approach assessed **(B)** proportions of hepatocytes, lymphocytes and myeloid cells in total liver cells per tissue section and **(C)** spatial distribution in representative liver samples. Regions with higher (red circles) and lower (green circles) myeloid cell densities were selected **(D)** Differentially generated metabolites were illustrated (higher myeloid cell infiltrated regions *vs.* lower myeloid cell infiltrated regions) in Sham, Sham + DR and SG groups. **(E)** Function enrichment of upregulated differentially generated metabolites (higher myeloid cell infiltrated regions *vs.* lower myeloid cell infiltrated regions) in the liver sample of SG group was analyzed based on the KEGG database. Abbreviations: C: control; Sham/S: sham surgery; DR: dietary restriction; SG: sleeve gastrectomy; KEGG: Kyoto encyclopedia of genes and genomes. The unpaired t-test, the Fisher's exact test and the one-way ANOVA test followed by Tukey's multiple comparison were performed. '*' represents '*p* < 0.05' and statistical significance.

**Figure 8:**
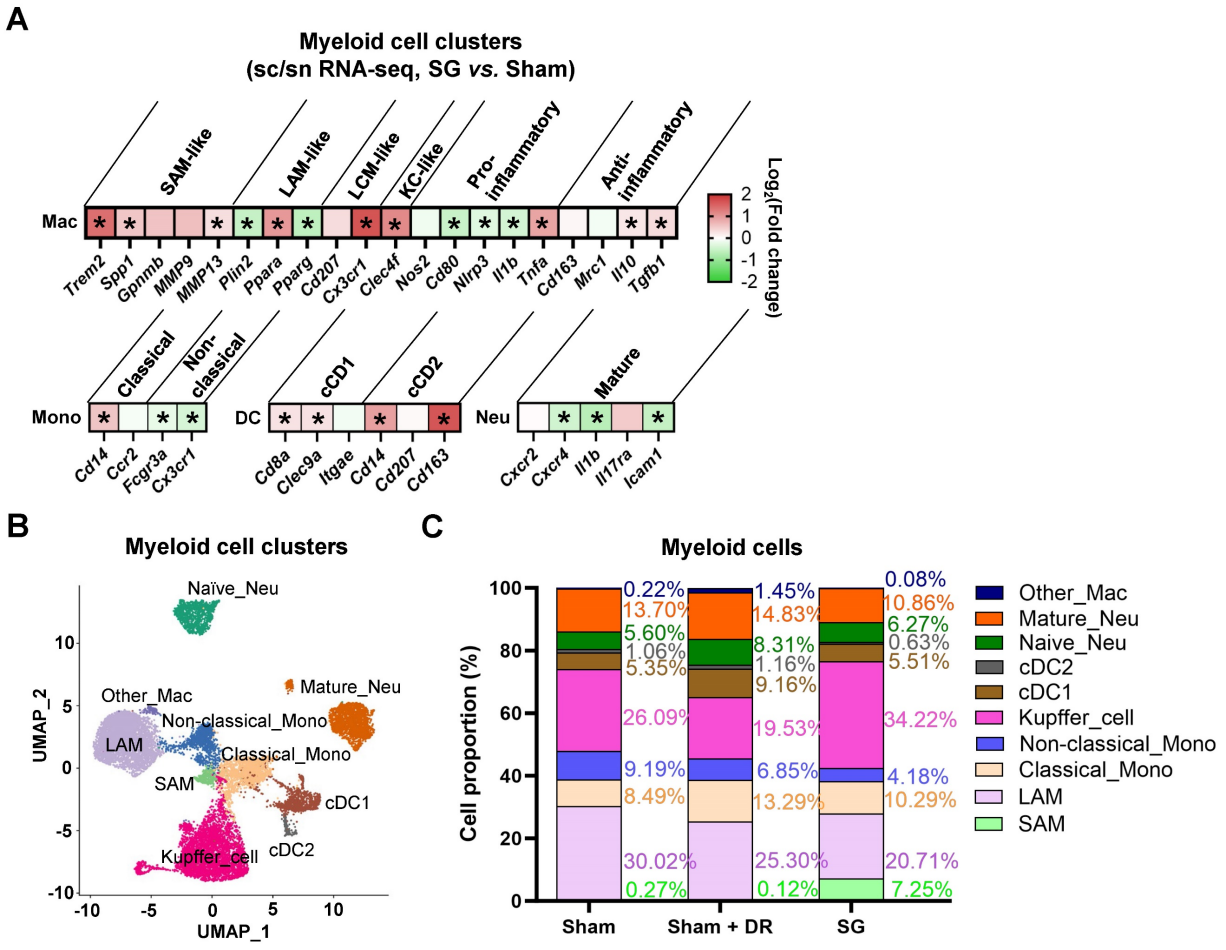
** Sleeve gastrectomy induces phenotypic alterations in myeloid cells. (A)** The expression of phenotype-associated markers in myeloid cell clusters was illustrated based on sc/sn RNA-seq data (SG *vs.* Sham). **(B)** Myeloid cells were characterized into phenotypic subtypes (SAM, LAM, non-/classical monocytes, KC, cDC1/2, mature/naïve neutrophils and other macrophages) with annotations. **(C)** The proportion of myeloid cell subtypes in Sham, Sham + DR and SG groups were illustrated. Abbreviations: Sham: sham surgery; DR: dietary restriction; SG: sleeve gastrectomy; sc/sn: single-cell/single-nuclei; Mono: monocytes; Mac: macrophages; (c)DC: (conventional) dendritic cells; Neu: neutrophils; SAM: scar-associated macrophage; LAM: lipid-associated macrophage; LCM: liver capsular macrophage; KC: Kupffer cell. The unpaired t-test and Fisher's exact test were performed. '*' represents '*p* < 0.05' and statistical significance.

AI-based pathological exploration has evolved as a promising and precise tool for MASLD investigations 19. Hence, in this study, a deep-learning model was established based on cell annotations (hepatocytes, lymphocytes and myeloid cells) that were supervised by liver pathology experts (Fig. 7A). Accordingly, the numbers of hepatocytes, lymphocytes and myeloid cells were assessed on a total of 18 rat liver samples. Importantly, hepatocyte proportions (70 - 80%) of total liver cells were not significantly altered by different interventions (Fig. 7B). Lymphocyte proportions were significantly increased in Sham with HFD (1.5 - 6.1%) *vs.* healthy control (0.05 - 2.1%) but declined upon Sham + DR (0.7 - 2.0%) and SG (0.5 - 2.2%) (*vs.* Sham) (Fig. 7B). Simultaneously, SG exacerbated the infiltration of myeloid cells (*vs.* healthy control, *vs.* Sham + DR) (Fig. 7B). In comparison, the numbers of lymphocytes and myeloid cells were assessed based on the sc/sn RNA-seq dataset. Myeloid cell proportions tended to increase by SG (Fig. S7A and B). Besides, the cell distribution of lymphocytes and myeloid cells was depicted to correspondingly map the metabolites in diverse cell regions (Fig. 7C). The abundance of total metabolites between regions with high or low myeloid cell infiltration was compared (high-/low-infiltration region selection displayed in Fig. 7C). SG remarkably increased metabolite abundance in regions with high myeloid cell infiltration (Fig. 7D). In addition, upregulated metabolites in regions rich in myeloid cells significantly refer to processes of autophagy, together with the metabolism of choline, linoleic acids, cholesterol and glycerophospholipids (Fig. 7E). Given the diverse phenotypic functions of macrophages in liver, a list of markers (*Trem2*, *Spp1*, *Gpnmb*, *Mmp9*, *Mmp13*, *Plin2*, *Ppara*, *Pparg*, *Cd207*, *Cx3cr1*, *Clec4f*, *Nos2*, *Cd80*, *Nlrp3*, *Il1b*, *Tnfa*, *Cd163*, *Mrc1*, *Il10* and *Tgfb1*) was selected from the sc/sn RNA-seq dataset to assess phenotypes of scar-associated macrophage (SAM), lipid-associated macrophage (LAM), liver capsular macrophage (LCM), Kupffer cell (KC), pro- and anti-inflammatory macrophages 20-22. Comparing macrophage clusters (SG *vs.* Sham), markers of SAM, LCM and KC were upregulated (SG *vs.* Sham), whereas pro-inflammatory macrophage markers were downregulated (SG *vs.* Sham and Sham + DR *vs.* Sham) (Fig. 8A, Fig. S7C). Simultaneously, SG promoted classical phenotypes of monocytes but suppressed neutrophil maturation. In contrast, DR tended to promote conventional DC (cDC) 1 phenotype and neutrophil maturation (Fig. 8A, Fig. S7C). In addition, myeloid cells were further divided into subtypes (SAM, LAM, non-/classical monocytes, KC, cDC1/2, mature/naïve neutrophils and other macrophages) by phenotypic characteristics and annotated (Fig. 8B). In line, proportions of myeloid cell subtypes were generated for Sham, Sham + DR and SG groups. Intriguingly, SG led to a lower frequency of LAM and mature neutrophils but a higher frequency of SAM and KC in comparison to Sham and Sham + DR (Fig. 8C). Taken together, SG and DR enhanced lipid metabolism in the liver, while SG promoted myeloid cell accumulation in the liver, facilitating metabolic improvement and liver repair processes. Nonetheless, SG may fuel fibrosis via enhanced SAM population in the liver.

### SG facilitates liver repair processes in steatotic livers

**Figure 9:**
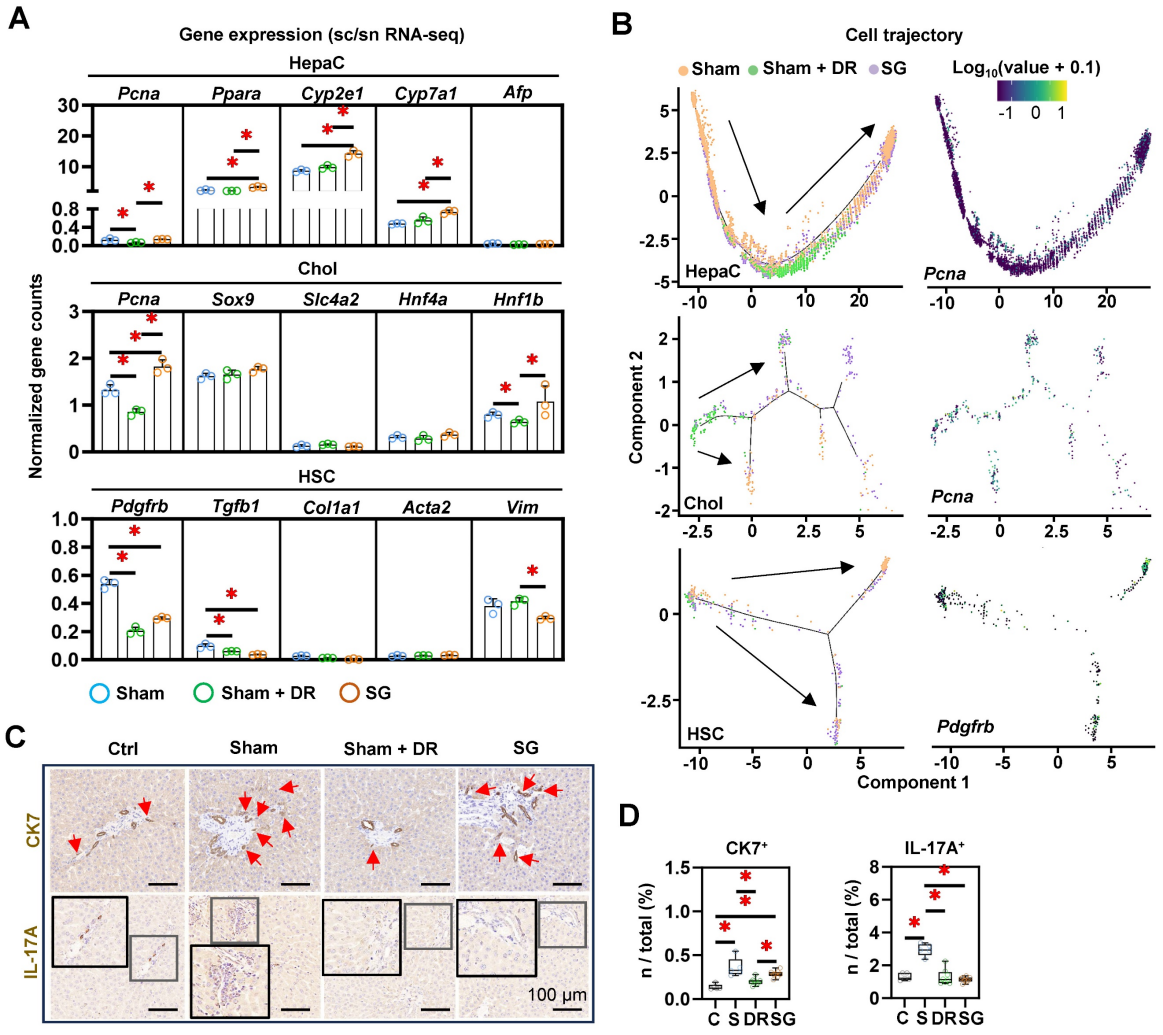
** Dietary restriction and sleeve gastrectomy alter the cellular differentiation of hepatocytes, cholangiocytes and hepatic stellate cells. (A)** Cellular characteristic markers of HepaC (*Pcna*, *Ppara*, *Cyp2e1*, *Cyp7a1* and *Afp*), Chol (*Pcna*, *Sox9*, *Slc4a2*, *Hnf4a* and *Hnf1b*) and HSC (*Pdgfrb*, *Tgfb1*, *Col1a1*, *Acta2* and *Vim*) were displayed. **(B)** Cell trajectories of HepaC, Chol and HSC together with *Cyp2e1* expression in HepaC, *Pcna* expression in Chol, and *Pdgfrb* expression in HSC. CK7- and IL-17A- expressing cells were **(C)** illustrated in situ and **(D)** quantified from total rat liver cells. Abbreviations: C: control; Sham/S: sham surgery; DR: dietary restriction; SG: sleeve gastrectomy; HepaC: hepatocyte; Chol: cholangiocyte; HSC: hepatic stellate cell; KEGG: Kyoto encyclopedia of genes and genomes. The one-way ANOVA test was performed. '*' represents '*p* < 0.05' and statistical significance.

The strong accumulation of macrophages in SG-treated livers, in conjunction with the weight development and metabolic improvement, suggested the induction of myeloid cell-driven hepatic repair processes 23. To further assess the cellular phenotypic alterations in the liver, a list of markers was explored (for hepatocytes: *Pcna*, *Ppara*, *Cyp2e1*, *Cyp7a1* and *Afp*; for cholangiocytes: *Pcna*, *Sox9*, *Slc4a2*, *Hnf4a* and *Hnf1b*; for HSCs: *Pdgfrb*, *Tgfb1*, *Col1a1*, *Acta2* and *Vim*) (Fig. 9A). The expression of *Pcna*, *Ppara*, *Cyp2e1* and *Cyp7a1* in hepatocytes, the expression of *Pcna* and *Hnf1b* in cholangiocytes significantly upregulated by SG (*vs.* Sham). In contrast, the expression of *Pcna* in hepatocytes and cholangiocytes in Sham + DR, and the expression of *Pdgfrb* in HSCs in Sham + DR and SG were downregulated (*vs.* Sham) (Fig. 8A). Besides, cell differentiation was assessed and depicted using a cell trajectory analysis model (Fig. 8B). Cell trajectory of hepatocytes implied higher expression of *Pcna*, *Cyp2e1* and *Ppara* in Sham + DR and SG groups than the Sham group, while the expression distinctly varied with the cell trajectory (Fig. 9B, Fig. S8). Cholangiocytes were classified into three groups. Cholangiocytes from Sham and SG groups were more associated with the expression of *Pcna* and *Slc4a2* but not with the expression of *Sox9* (Fig. 9B, Fig. S8). Furthermore, HSCs of Sham + DR groups were shown in relatively inactivated clusters associated with low *Pdgfrb* expression (Fig. 9B), but not associated with the expression of *Tgfb1* and *Col1a1* (Fig. S8). In addition, IHC analyses revealed the occurrence of ductular reaction by immunostaining of CK7, and interleukin (IL)-17A^+^ cells, typically associated with liver fibrosis 24, 25. The presence of CK7^+^ cells was significantly increased by steatosis induction, while it was alleviated by Sham + DR (*vs.* Sham) (Fig. 9C and D). Simultaneously, the presence of IL-17A^+^ cells was significantly increased by HFD but not in the Sham + DR and SG (*vs.* Sham) (Fig. 9C and D). Hence, our data indicated that Sham + DR and SG can attenuate the induction of fibrogenic immune response, while Sham + DR repressed ductular reaction. Taken together, SG and especially DR tend to reduce the involvement of portal area injury (particularly ductular reaction and fibrogenesis). Notably, DR appears to compromise regenerative capacities in hepatocytes, which requires further investigation.

### DR affects zonation characteristics of periportal hepatocytes

**Figure 10:**
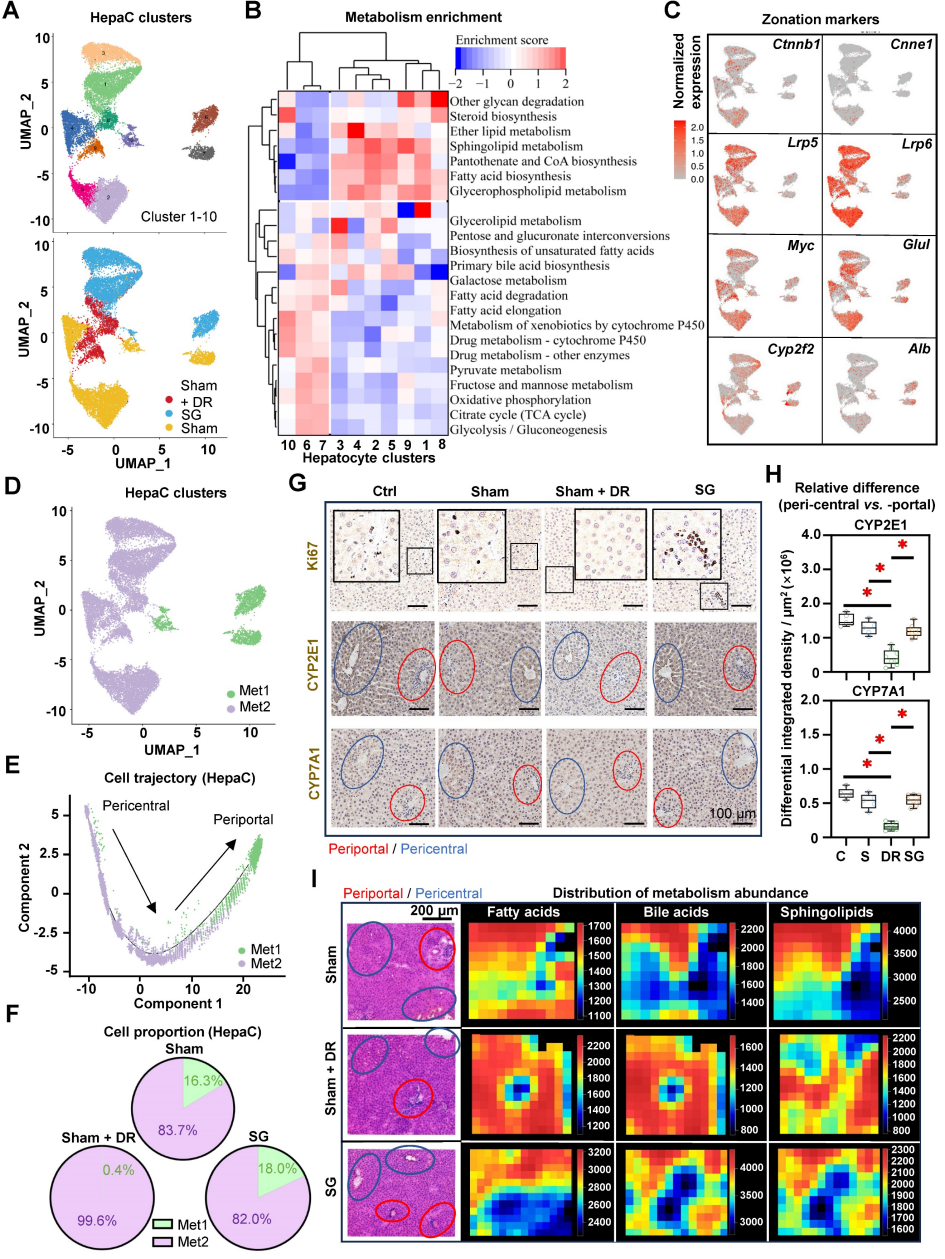
** Dietary restriction compromises zonation characteristics of periportal hepatocytes. (A)** Subclusters of rat hepatocytes from Sham, Sham + DR and SG are illustrated in UMAPs. **(B)** Metabolism enrichment on each hepatocyte subcluster is displayed in a matrix. **(C)** Gene expression of *Ctnnb1*, *Cnne1*, *Lrp5*, *Lrp6*, *Myc*, *Glul*, *Cyp2f2* and *Cyp7a1* illustrated in rat hepatocytes. **(D)** Metabolism group-1 and -2 in rat hepatocytes. **(E)** Metabolism group-1 and -2 in the cell trajectory of rat hepatocytes. **(F)** Proportions of metabolism group-1 and -2 in rat hepatocytes of Sham, Sham + DR and SG groups. **(G)** Protein levels of Ki67, CYP2E1 and CYP7E1 illustrated in rat liver tissue (each view contains one or more portal and central areas) of Sham, Sham + DR and SG groups. **(H)** Relative differences of CYP2E1 and CYP7E1 levels between peri-central and -portal regions in each rat liver tissue of Sham, Sham + DR and SG groups. **(I)** Spatial distribution of fatty acid, bile acid and sphingolipid metabolites. In H&E- and IHC-characterized rat liver sample sections, periportal regions were outlined in red circles and pericentral regions were outlined in blue circles. Abbreviations: Sham: sham surgery; DR: dietary restriction; SG: sleeve gastrectomy; HepaC: hepatocyte; Met: metabolism; CYP: cytochrome. H&E: hematoxylin and eosin. '*' represents '*p* < 0.05' and statistical significance.

Given the remarkable cell trajectory with variable expression of phenotypic markers, differential characteristic alterations in hepatocyte subtypes were hypothesized. Hence, hepatocytes were further dissected into subpopulations based on the sc/sn RNA-seq dataset (Fig. 10A). Metabolism-related function enrichment analysis revealed two distinct groups: metabolism one group (Met1, including cluster 6, 7 and 10) enriched metabolism processes of glycolysis, lipids and drugs, while metabolism two group (Met2, including clusters 1, 2, 3, 4, 5, 8 and 9) enriched fatty acid biosynthesis (Fig. 10B).

Interestingly, illustrations of the gene expression (pericentral hepatocyte markers: *Ctnnb1*, *Ccne1*, *Lrp5*, *Lrp6*, *Myc* and *Glul*; periportal hepatocyte markers: *Cyp2f2* and *Alb*) further identified that the Met1 group harbors more periportal hepatocytes, while the Met2 group harbors more pericentral hepatocytes (Fig. 10C and D). Furthermore, cell trajectory analysis indicated the explicit differentiation status of these two metabolism groups, which supports the zonal classification (Fig. 10E). In comparison to periportal (Met1) hepatocytes, pericentral (Met2) hepatocytes illustrated higher numbers and strength of interactions with immune cells, while myeloid cells, T lymphocytes and NK cells exerted particularly high involvement in cellular interactions (Fig. S9A and B). Particularly, periportal phenotypes accounted for 15 - 20% of total hepatocytes in both Sham and SG groups but were almost absent (0.4%) in the Sham + DR group, indicating a phenotypic reshaping of hepatocytes induced by DR (Fig. 10F). In addition, metabolism markers [cytochrome (CYP)2E1 and CYP7A1] and cell proliferation (Ki67) were investigated in situ (Fig. 10G). Strikingly, proliferative cells (Ki67^+^) were more present in the liver tissues of the SG group than in other groups, indicating a strong potential for liver regeneration led by SG (Fig. 10G). CYP2E1 and CYP7A1 were determined to be more enriched surrounding central veins in Ctrl, Sham and SG groups, but to be homogeneously distributed in Sham + DR groups (Fig. 10G and H). In addition, the spatial distribution of fatty acid, BA and sphingolipid metabolites appeared to be enriched around periportal areas in Sham and SG groups but rather widely spread in Sham + DR groups (Fig. 10I). Taken together, DR deprives hepatocyte zonation features potentially by reshaping periportal hepatocyte characteristics, which may compromise the capacities of metabolism and regeneration in steatotic livers in comparison to SG.

## Discussion

In this study, we employed multiomics methods to unravel relevant alterations of obesity-related parameters and liver steatosis following SG and DR in both patient cohorts and rat models. Specifically, sc/sn RNA-seq data unveiled an augmentation of lipid metabolism in both hepatocytes and immune cells, with a notable increase in myeloid cells following SG. This data was supported by spatially-resolved metabolomics analysis that revealed the strengthened metabolic capacities of liver cells induced by SG and DR. Notably, SG elicited elevated lipid metabolic capacities in regions exhibiting intense myeloid cell infiltration, underscoring the intricate interplay between metabolic and immune responses in the liver microenvironment. PPAR-α emerged as a prominent candidate mediating the observed metabolic enhancements induced by both SG and DR. Additionally, the increased serological levels of bile acids and gut-derived FGF-19, together with hepatic butanoate metabolism underscored the putative pivotal roles of the gut-liver axis in modulating liver metabolism post-SG.

To date, only a few studies have employed the bulk transcriptomic analysis of patients' liver samples post-BS to identify alterations of inflammation and lipid metabolism, yet those studies suggested potential therapeutic approaches for MASLD 26. Essentially, PPARα was elucidated as a key nuclear receptor responsible for weight-loss-induced MASLD amelioration 27, 28. Consequently, PPAR agonism has been regarded as a promising approach to tackling MASLD 29, 30. Robust preclinical data have determined the potential of targeting PPARα to improve lipid metabolism, inflammation, and fibrosis in MASLD 31, 32. In line, our previous study has proved that lanifibranor (a pan-PPAR agonist) significantly improves liver steatosis and fibrosis in mice and in an in-vitro liver-on-a-chip model of MASLD 33. However, numerous PPAR dual/pan agonists have failed to reach clinical practices, because they could not satisfy clinical endpoints (amelioration of e.g., liver injury, fibrosis, steatosis) 34. In addition, SG effectively enhanced the PPARα level and metabolic pathways broadly on hepatic parenchymal and immune cells, to a higher degree than DR. It has been demonstrated that PPARα activation in the liver can ameliorate the accumulation of circulating monocytes upon DR intervention 35. In addition, cell types were predicted from hematoxylin-eosin-stained rat liver sections using an AI-assisted digital method, regarded as an advanced tool for MASLD diagnosis 19, 36. Intriguingly, in rat MASLD models, SG can strengthen the macrophage pool despite a broad PPARα activation in the liver. Further characterization of the macrophage population indicates that SG endows the anti-inflammatory and repair-associated potential of macrophages rather than their detrimental pro-inflammatory roles in the liver.

It has been reported that lipid metabolism is critical in immune response during steatotic liver diseases 37-39. Accordingly, our study outlined plausible direct links between metabolism restoration and myeloid cell infiltration in post-SG steatotic rat livers. The diverse and contradictory roles of liver macrophages make them able to promote liver homeostasis, disease progression and remission depending on a plethora of possible phenotypic activation profiles 30, 40-42. Liver macrophages can mediate hepatic lipid metabolism during steatotic stress 43, 44, while lipid metabolism is closely associated with phenotypic alterations in macrophages 45, 46. Thus, the liver macrophage compartment remains an intriguing target for MASLD therapies 47-49. Moreover, the functions of liver macrophages vary distinctly by spatial distribution 50. In this study, we revealed that upon SG, the liver regions with high myeloid cell infiltration exert strong lipid metabolism capacity (e.g., autophagy and choline metabolism), while the causes are poorly understood. As reported, autophagy and choline metabolism play crucial roles in mediating myeloid cell (especially macrophage) phenotypes. For instance, lipid autophagy (term 'lipophagy') can potentiate their activation towards macrophage foam cells and a lipid-associated macrophage phenotype 51. Contrastingly, choline uptake and metabolism modulate macrophage inflammasome processes and promote a pro-inflammatory phenotype 52. Therefore, we hypothesize that SG-stimulated liver myeloid cells could better sense and assist metabolic stress in the liver, thereby leading to their heterogeneous distribution in the liver.

The hepatic metabolic and regenerative functions vary by hepatocyte zonation (periportal and pericentral), leading to diverse responses to injuries and therapies 53, 54. Emerging studies demonstrated that absent metabolic function in the periportal zone provokes cholestasis and ductular reaction 55, 56. On the other hand, compromised hepatic periportal characteristics may be attributed to dysregulated WNT signaling and cell regeneration 55, 57. Nowadays, the advances in spatially resolved multiomics approaches (e.g., sc/sn RNA-seq, spatial metabolomics, and spatial proteomics) provide both broader views and deeper understandings of heterogenic pathomechanisms of liver diseases 53, 58, 59. Our study elucidated that hepatic periportal characteristics were dramatically subsided by DR but preserved by SG. Since DR is rarely associated with adverse events in the liver, we hypothesized that periportal hepatocytes might acquire 'pericentral' features to aid hepatic metabolism. Previous studies have shown that fasting can influence hepatocyte zonation features, potentially by altering endoplasmic reticulum (ER)-mitochondria function in pericentral and mild-lobular hepatocytes 55, 60. However, a causative link between liver zonation and dietary intake or surgical interventions remains unknown. Likely, enhanced immunometabolism levels may interpret the preservation of zonal features upon the SG intervention. Furthermore, both DR and SG appeared to ameliorate stress in the liver portal area, illustrating less presence of ductular cells and fibrosis-promoters. In addition, our data indicated that the regenerative potentials of hepatocytes were enhanced by SG but not DR, which strengthened the findings from the latest study 18.

As observed in multiple clinical studies, FGF19 levels were increased in patients' circulation post-BS, thereby implying a potential regulatory mechanism of BS 61. Intriguingly, we also spotted that female patients tended to gain a higher FGF-19 increase than male patients post-SG. However, it has to be verified based on future studies with larger cohorts. Noteworthy, our study determined improved capacities of BA secretion and butanoate metabolism, as well as elevated FGF19 levels in serum upon the SG, suggesting that SG influences liver metabolism by regulating the gut-liver axis. The current understanding of MASLD pathogenesis is deficient in regard to insights into gut-liver crosstalk. This encompasses a number of key areas, including hepatic cell metabolism, inflammation, fibrogenesis, and complex cellular interactions, which play pivotal roles in MASLD progression 23, 30, 62. Increasing evidence indicates critical roles that BS may play in regulating the gut-liver axis, including GM, and BA metabolism 63. As suggested by recent studies, post-BS changes in the GM and BA circulation, as well as a decrease in the portal influx of free fatty acids, are beneficial to steatotic livers and obesity 64-66. Furthermore, BS can drastically repopulate GM and reverse the primary/secondary BA ratio in the intraluminal ileal, inducing improvement in metabolic syndrome in patients with MASLD 67. On the other hand, the butanoate production by GM was reported to be associated with bile acids and to mediate liver metabolism 68. Notably, butanoate/butyrate production can be enhanced after bariatric surgery 69, while favorable effects of butyrate have been proved on MASLD, including attenuating hepatic steatosis and inflammation while enhancing insulin sensitivity 70. Given the limited amount of literature focusing on the connection between PPAR-α, FGF-19, and butanoate metabolism, it is suggested that butyrate can upregulate PPAR-α in hepatocytes, thereby alleviating HFD-induced MASLD in rats through the activation of β-oxidation and inflammation suppression 71. Our study not only supports the fundamental roles of the gut-liver axis and gut-derived signals in protecting against liver steatosis, but also reveals disease ameliorations led by BS in contrast to simple DR.

Nonetheless, the limitations inherent in the current study should be acknowledged. The exclusion of patients with MASLD and obesity who solely underwent DR stemmed from challenges associated with dietary normalization to SG and inherent biases in dietary quantification. Furthermore, the sample size utilized for spatial metabolomics analysis was relatively modest, potentially constraining the generalizability of the findings. In addition, the *in vitro* modeling of BS remains challenging, while it is promising to employ multicellular systems (e.g., liver-on-a-chip 72) to break down mechanisms of immunometabolism in MASLD. Despite these limitations, this study highlights a prominent enhancement in liver immunometabolism upon SG. Notably, multi-omics approaches offer a comprehensive exploration of the cellular and metabolic landscape within steatotic livers, marking a pioneering endeavor in this domain. Beyond traditional analyses, the incorporation of sc/sn RNA transcriptome and spatially resolved metabolite distribution analyses provides unprecedented insights into the immunometabolism dynamics of MASLD post-DR and -SG.

## Materials and Methods

### Human cohort

The clinical retrospective study was conducted on 18 patients who received SG in The Third Affiliated Hospital of Nanjing Medical University/Changzhou Second People's Hospital (from January 2023 to July 2023). This cohort comprised n = 8 male (44.4%) and n = 10 female (55.6%) individuals at a median age of 34 years (range: 29 - 40). We included patients that fulfilled the following criteria: 1) BMI > 32.5 kg/m^2^; 2) Age: 16 - 65; 3) clinically diagnosed with MASLD (based on cardiometabolic risk factors and presence of steatosis on liver imaging; 4) diagnosed with type 2 diabetes or sleep apnea syndrome; 5) without a history of other hepatic or genetic illness; 6) received sleeve gastrectomy, without postoperative complications. Clinical indexes (e.g., BMI, serological indexes) were evaluated on the day before SG and 6 months post-surgery. This clinical retrospective study was approved by the ethics committee of The Third Affiliated Hospital of Nanjing Medical University/Changzhou Second People's Hospital (ID: [2023]KY124-01).

### Animal models and surgical interventions

Male wild-type SPRAGUE DAWLEY® rats aged 8 weeks were obtained from Laboratory Animal Co., Ltd. (Changzhou, China). Rats were bred under specific-pathogen-free conditions and fed with HFD (D12492, Research Diets, Inc., USA) for 12 weeks. Five rats received sham surgery followed by uncontrolled HFD, 9 rats received sleeve gastrectomy followed by uncontrolled HFD, and 8 rats received sham surgery followed by restricted HFD feeding (comparable to dietary intake of post-SG rats) for 8 weeks. Procedures of sleeve gastrectomy were illustrated in supplementary data (Supplementary methods and Fig. S1) and were based on our previous research 73. Five healthy control rats were set with normal diet feeding but without any specific interventions. All these procedures were performed and supported by Kerbio Co., Ltd., Changzhou, China. Animal experiments were approved by the Institutional Animal Care and Use Committee (ID: IACUC23-0068).

### Serological tests

Patients' blood samples were acquired from the median cubital vein. Rat blood samples were harvested through rat hearts immediately after rats were sacrificed. Serum was harvested using centrifugation (450×g, 10 min), and stored at -80℃. Circulating markers including AST, ALT, PPG, γ-GT, TG, cholesterol (CHO), HDL-C, LDL-C, alkaline phosphatase (ALP) and TBA were measured using Indiko analyzer (ThermoFisher, USA). Cytokines including IL-6, IL-10, FGF-19, FGF-21 and transferrin (TRF) were measured using ELISA kits and a microplate reader (Rayto, China). All measurements were conducted following the manufacturer's instructions. Antibodies that were used in this study are listed in Table S1.

### Immunohistochemistry and histological exploration

Liver tissues were harvested immediately after rats were sacrificed. Tissues were fixed in 10%-formalin for 24 hours, followed by paraffin embedding. Sections of 5 µm thickness were obtained using a HistoCore BIOCUT microtome (Leica, Germany). Hematoxylin and eosin (HE) staining were performed using standard procedures. Extracellular collagen area was indicated using the Picro Sirius Red Stain Kit (Abcam, UK). The IHC method was employed using the kits purchased from Beyotime Biotechnology, China. Antigen retrieval was conducted using a heat-induced epitope retrieval method within a citrate buffer. Primary antibodies against the target proteins were applied, followed by appropriate secondary antibodies. Diaminobenzidine (DAB) was used for chromogenic detection, and counterstaining was performed with hematoxylin. Slides were examined under a microscope to evaluate protein localization and expression levels. In situ staining was observed under a light microscope (Olympus, Japan). Antibodies used in this study are listed in Table S1. For the quantitation of cell nucleus and protein levels, FIJI software was utilized 74. In particular, relative staining densities of proteins per µm2 were assessed and compared between peri-central and -portal regions in each tissue slide.

### Single-cell and single-nuclei sequencing and analysis

Single cells and cell nuclei were isolated and extracted from freshly collected rat liver samples using the dissection kit purchased from OE Biotech Co., Ltd., Shanghai, China. The libraries were acquired following the manufacturer's protocol and then sequenced using the MobiNova platform (Shanghai, China). The integration of sc/sn-RNA sequencing data was operated following the common protocol with eliminating batch effects 75. Cell Ranger was used to filter the data, while the downstream analyses were conducted using Seurat (V5.0.1) 76, CellChat (1.6.0) 77, and Monocle2 78 software packages for R. Cell clusters were annotated based on the expression of representative cell makers and verified using the Enrichr database 79. Function enrichment was performed based on Gene ontology (GO) and Kyoto Encyclopedia of Genes and Genomes (KEGG) databases. All the technical procedures including sampling, quality control, library build-up and sequencing analysis were supported by the genome platform of OE Biotech Co., Ltd., Shanghai, China.

### Spatial metabolomics analysis

Liver samples were freshly acquired from rats and then frozen at -80℃ immediately after optimal cutting temperature (OCT) embedding. Embedded tissue samples were sliced into 5 µm using a cryo-microtome CM 1950 (Leica, Germany). In-situ profiles of total metabolites were detected with positive and negative ion binding approaches using the MSI metabolomics method 80. Tissue regions were annotated after H&E staining. Metabolite enrichment was assessed based on the KEGG database. All the technical procedures including sample preparation, quality control, detection and analysis were supported by the metabolomics platform of Luming Biotechnology Co., Ltd. Shanghai, China.

### AI-guided imaging analysis

The AI-guided imaging analysis performed herein is based on a previously published approach 81-83. H&E-stained rat liver sections were scanned using a light microscope (Olympus, Japan). Processing of the whole slide images (WSIs) was operated using QuPath 0.4.4 following a pre-defined protocol 84. Sparse annotations, comprising approximately 15 cells for each of the cell populations, were manually labeled across three WSIs representing different conditions (Sham, Sham + DR and SG). A simple artificial neural network classifier with two hidden layers, consisting of 20 and 10 neurons, respectively, was trained to distinguish between the three classes versus the rest. Example regions without these classes were used as negative examples during training. Subsequently, cell classification and measurements were exported, and for each cell population, 2D hexagonal binning plots of the number of cells were generated in Python (using the matplotlib function 'hexbin') 85. The model was trained for representative cells (at least 15 cells/sample' of hepatocytes, lymphocyte-like cells and myeloid-like cells) based on three liver tissue slides, which were defined with the assistance of pathological expertise. The trained model was applied to the sample cohort of rat liver sections (including 5 Ctrl, 5 Sham, 8 Sham + DR and 8 SG samples). Detailed methodology is described in the Supplementary methods.

### Statistical analysis

The GraphPad Prism 9.0 software (GraphPad Software, USA) and the ggplot2 package (version: 3.4.4) for R were used to generate plots 86. The statistical tests used in this study are indicated in the figure legend for each panel. Data are presented as the mean ± S.D. '*p* < 0.05' was considered a significant difference.

## Supplementary Material

Supplementary methods, figures and tables.
