# Supplementary material for: Multiomics analyses decipher intricate changes in the cellular and metabolic landscape of steatotic livers upon dietary restriction and sleeve gastrectomy
Source: Int J Biol Sci. 2024 Aug 19;20(11):4438–57. doi: 10.7150/ijbs.98362 (PMC11380448; doi:10.7150/ijbs.98362)
Supplement: Supplementary file 1 — Supplementary methods, figures and tables. [file ijbsv20p4438s1.pdf]

Supplementary Text

Supplementary methods

Animal modeling and surgery procedures

SPRAGUE DAWLEY® male rats, aged 8 weeks, were purchased from Cavens Laboratory Animal Co., Ltd. After one week of adaptation feeding, they were fed with a high-fat diet (HFD; D12492, Research Diets, Inc., USA) for 12 weeks. They were randomly divided into three treatment groups: sleeve gastrectomy (SG), sham surgery, and sham surgery + diet restriction (DR). Before the surgery, the rats were fasted and deprived of water for 12 hours. They were then anesthetized with 1% pentobarbital sodium (4 mL/kg) intraperitoneal injection, placed on a fixed board, and the surgical area was disinfected. An incision of about 2 cm was made in the middle of the abdomen, and the stomach was located and pulled out from the abdominal cavity. Subsequently, the hepatic and splenic ligaments were ligated and sectioned, and the short gastric and gastrosplenic vessels were ligated. A pair of vascular forceps was applied from the cardia HIS Angle along the greater curvature of the stomach. The gastric body was quickly cut off with tissue scissors, and the incision of the gastric cavity was sutured continuously with 5 - 0 number sterile thread. The vascular clamp was then used to achieve control over the stomach, extending from the incision up to 3 mm above the pylorus. Approximately 70% to 80% of the gastric tissue, including the fundus, was excised, and subsequently, the incision was closed through continuous suturing. Finally, a varus suture was performed to minimize the risk of postoperative wound infection. The peritoneum, muscle, and skin were meticulously closed in layers to complete the sleeve gastric operation. Intraperitoneal injection of Meloxicam at a dosage of 0.5 mg/kg was administered for pain management on the first day after surgery (sham and SG). Additionally, rats preemptively received Penicillin at a dosage of 30,000 U/kg on the first day after surgery (sham and SG). Following the surgery, all rats underwent a one-day fasting period before being provided with sugar and saline on the second day post-operation. Subsequently, their diet gradually turned back to planned feeding.

The anesthesia and perioperative treatment of the sham group and sham + DR group were the same as that of the SG group. An incision of 1 cm was simply made in the stomach and sutured in place for the sham group. Additionally, the diet of the sham + DR group matched that of the SG group. The daily food intake of the SG group was measured, and the average amount was fed to the sham + DR group on the following day. The whole procedure is described in Figure S1.

Artificial intelligence (AI)-guided imaging analysis

The whole slide images (WSIs) with a magnification of 20x (0.2749 μm/pixel) were processed using QuPath 0.4.4 to measure hepatocyte, lymphocyte, and myeloid cell populations. Tissue detection was conducted by applying a threshold of 200 on the average of the red, green, and blue channels at a resolution of 12.5 μm/pixel on the smoothed image, followed by median filtering and smoothed coordinates. Tissue regions detected smaller than 1,000 px and holes smaller than 500 px were subsequently removed.

Nuclei were identified based on the hematoxylin optical density at a resolution of 1.5 μm/pixel, using a background radius of 8 and a sigma of 1.5. Following the opening morphological operation, objects detected smaller than 10 μm^2 or larger than 500 μm^2 were excluded from further analysis. The intensity threshold was set to 0.1, with a maximum background intensity of 2. Additionally, the boundaries of the detected nuclei were smoothed.

Sparse annotations, comprising approximately 15 cells for each of the cell populations, were manually labeled across three WSIs representing different conditions (Sham, DR and SG). A

simple artificial neural network classifier with two hidden layers, consisting of 20 and 10 neurons, respectively, was trained to distinguish between the three classes versus the rest. Example regions without these classes were used as negative examples during training. Subsequently, cell classification and measurements were exported, and for each cell population, 2D hexagonal binning plots of the number of cells were generated in Python (using matplotlib function 'hexbin') with a blue-yellow-red color map for visualization purposes. The reported metrics included total cell counts of each cell type, as well as the ratio of each cell type to both the total tissue area and the total number of detected cells.

An analogous workflow was applied to 27 test WSIs. Considering slight color differences, the tissue detector utilized a threshold of 240 at a resolution of 20 μm/pixel, removing tissue regions smaller than 5,000 μm^2 and holes smaller than 1000 px^2. The same cell detector, cell classifier, and post-analysis procedures were employed for consistency.

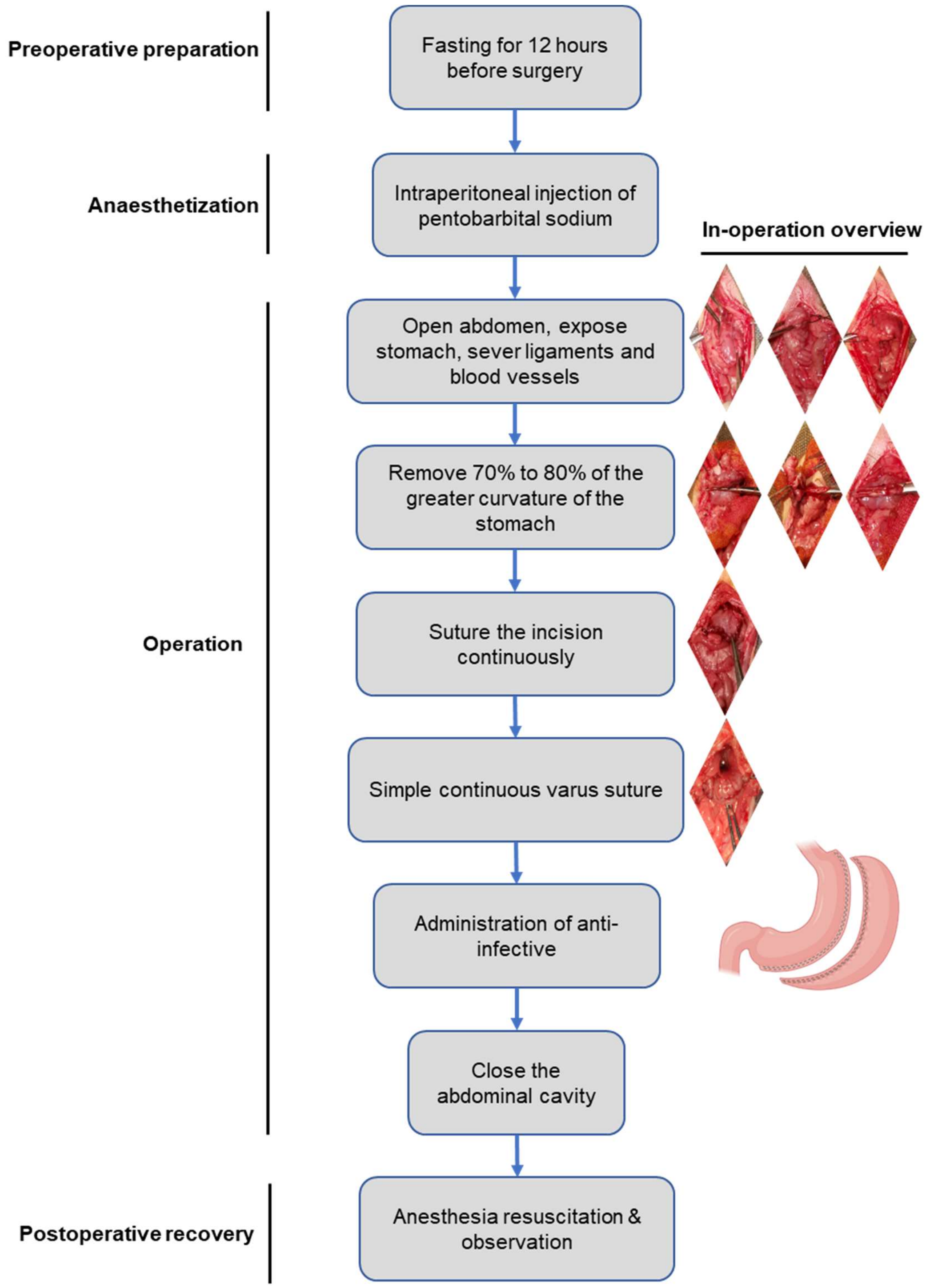

Fig. S1. Technical procedures of sleeve gastrectomy on rats.

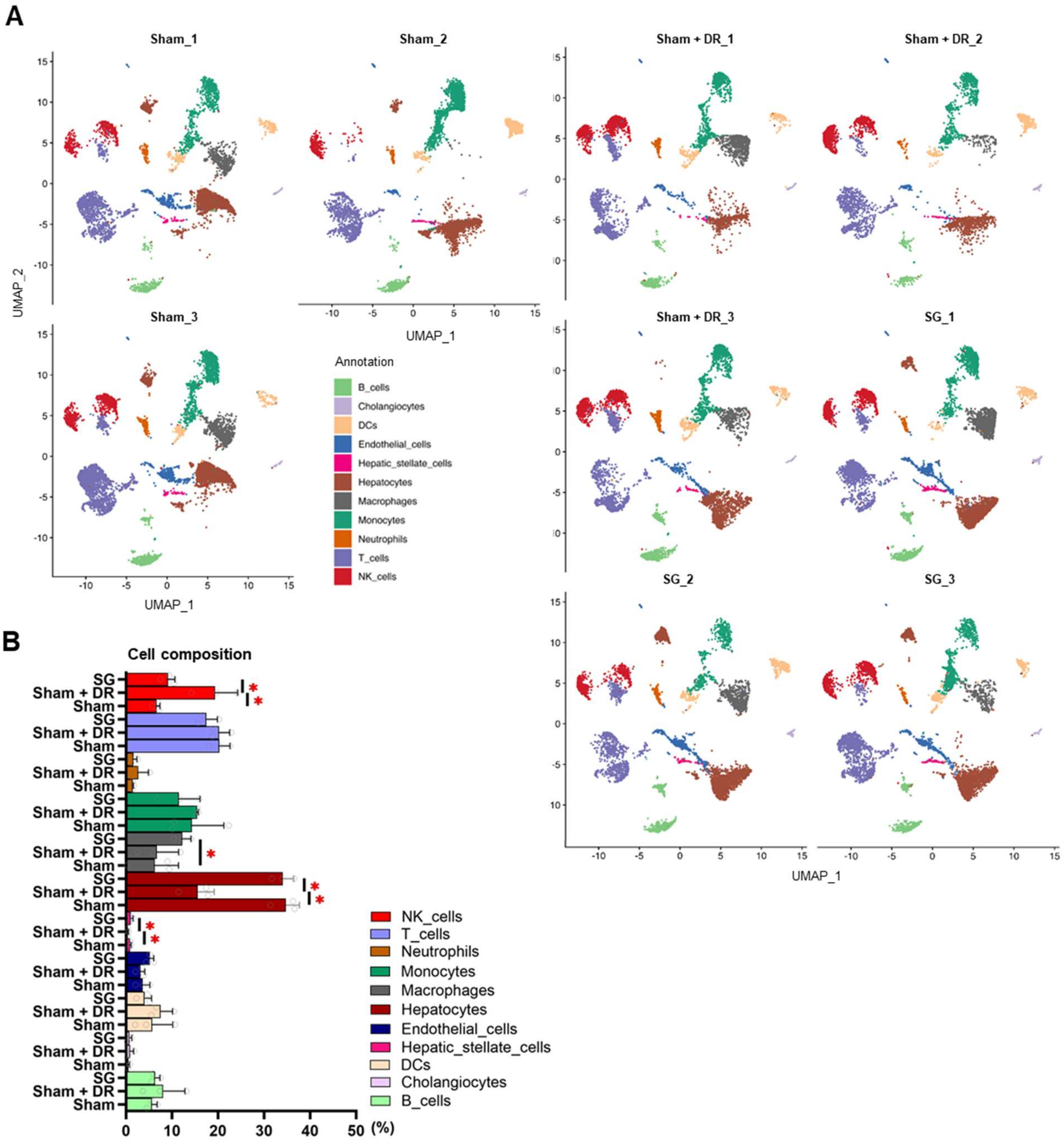

Fig. S2. Single-cell/single-nuclei RNA-seq analysis identifies cell populations. (A) UMAPs illustrating the cell distribution of all individual samples upon Sham, Sham + DR and SG. **(B)** Histograms illustrating cell proportions of all individual samples upon Sham, Sham + DR and SG. Abbreviations: Sham: sham surgery; DR: dietary restriction; SG: sleeve gastrectomy; B cells: B lymphocytes; T_cells: T lymphocytes; DC: dendritic cell.

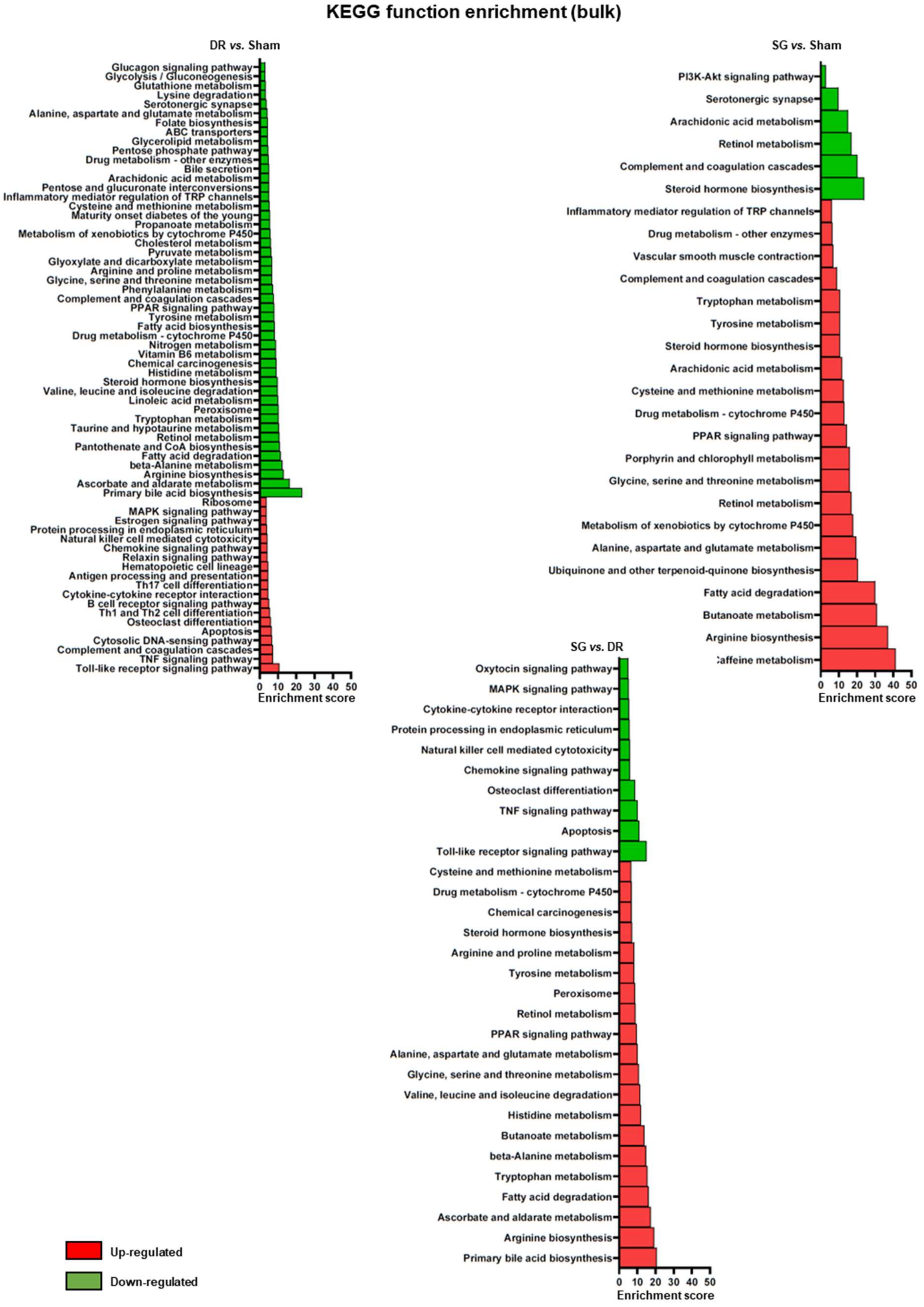
KEGG function enrichment (bulk)
DR vs. Sham
Glucagon signaling pathway
Glycolysis / Gluconeogenesis
Glutathione metabolism
Lysine degradation
Serotonergic synapse
Alanine, aspartate and glutamate metabolism
Folate biosynthesis
ABC transporters
Glycerolipid metabolism
Pentose phosphate pathway
Drug metabolism - other enzymes
Bile secretion
Arachidonic acid metabolism
Pentose and glucuronate interconversions
Inflammatory mediator regulation of TRP channels
Cysteine and methionine metabolism
Maturity onset diabetes of the young
Propanoate metabolism
Metabolism of xenobiotics by cytochrome P450
Cholesterol metabolism
Pyruvate metabolism
Glyoxylate and dicarboxylate metabolism
Arginine and proline metabolism
Glycine, serine and threonine metabolism
Phenylalanine metabolism
Complement and coagulation cascades
PPAR signaling pathway
Tyrosine metabolism
Fatty acid biosynthesis
Drug metabolism - cytochrome P450
Nitrogen metabolism
Vitamin B6 metabolism
Chemical carcinogenesis
Histidine metabolism
Steroid hormone biosynthesis
Valine, leucine and isoleucine degradation
Linoleic acid metabolism
Peroxisome
Tryptophan metabolism
Taurine and hypotaurine metabolism
Retinol metabolism
Pantothenate and CoA biosynthesis
Fatty acid degradation
beta-Alanine metabolism
Arginine biosynthesis
Ascorbate and aldarate metabolism
Primary bile acid biosynthesis
Ribosome
MAPK signaling pathway
Estrogen signaling pathway
Protein processing in endoplasmic reticulum
Natural killer cell mediated cytotoxicity
Chemokine signaling pathway
Relaxin signaling pathway
Hematopoietic cell lineage
Antigen processing and presentation
Th17 cell differentiation
Cytokine-cytokine receptor interaction
B cell receptor signaling pathway
Th1 and Th2 cell differentiation
Osteoclast differentiation
Apoptosis
Cytosolic DNA-sensing pathway
Complement and coagulation cascades
TNF signaling pathway
Toll-like receptor signaling pathway
0 10 20 30 40 50
Enrichment score
SG vs. Sham
PI3K-Akt signaling pathway
Serotonergic synapse
Arachidonic acid metabolism
Retinol metabolism
Complement and coagulation cascades
Steroid hormone biosynthesis
Inflammatory mediator regulation of TRP channels
Drug metabolism - other enzymes
Vascular smooth muscle contraction
Complement and coagulation cascades
Tryptophan metabolism
Tyrosine metabolism
Steroid hormone biosynthesis
Arachidonic acid metabolism
Cysteine and methionine metabolism
Drug metabolism - cytochrome P450
PPAR signaling pathway
Porphyrin and chlorophyll metabolism
Glycine, serine and threonine metabolism
Retinol metabolism
Metabolism of xenobiotics by cytochrome P450
Alanine, aspartate and glutamate metabolism
Ubiquinone and other terpenoid-quinone biosynthesis
Fatty acid degradation
Butanoate metabolism
Arginine biosynthesis
affeine metabolism
0 10 20 30 40 50
Enrichment score
SG vs. DR
Oxytocin signaling pathway
MAPK signaling pathway
Cytokine-cytokine receptor interaction
Protein processing in endoplasmic reticulum
Natural killer cell mediated cytotoxicity
Chemokine signaling pathway
Osteoclast differentiation
TNF signaling pathway
Apoptosis
Toll-like receptor signaling pathway
Cysteine and methionine metabolism
Drug metabolism - cytochrome P450
Chemical carcinogenesis
Steroid hormone biosynthesis
Arginine and proline metabolism
Tyrosine metabolism
Peroxisome
Retinol metabolism
PPAR signaling pathway
Alanine, aspartate and glutamate metabolism
Glycine, serine and threonine metabolism
Valine, leucine and isoleucine degradation
Histidine metabolism
Butanoate metabolism
beta-Alanine metabolism
Tryptophan metabolism
Fatty acid degradation
Ascorbate and aldarate metabolism
Arginine biosynthesis
Primary bile acid biosynthesis
0 10 20 30 40 50
Enrichment score
Up-regulated
Down-regulated

Fig. S3. Single-cell/single-nuclei RNA-seq analysis deciphers differentially expressed genes. KEGG function enrichment analysis on significant DEGs upon comparisons (Sham + DR *vs.* Sham and SG *vs.* Sham). Abbreviations: Sham: sham surgery; DR: dietary restriction; SG: sleeve gastrectomy; KEGG: Kyoto Encyclopedia of Genes and Genomes; DEG: differentially expressed gene.

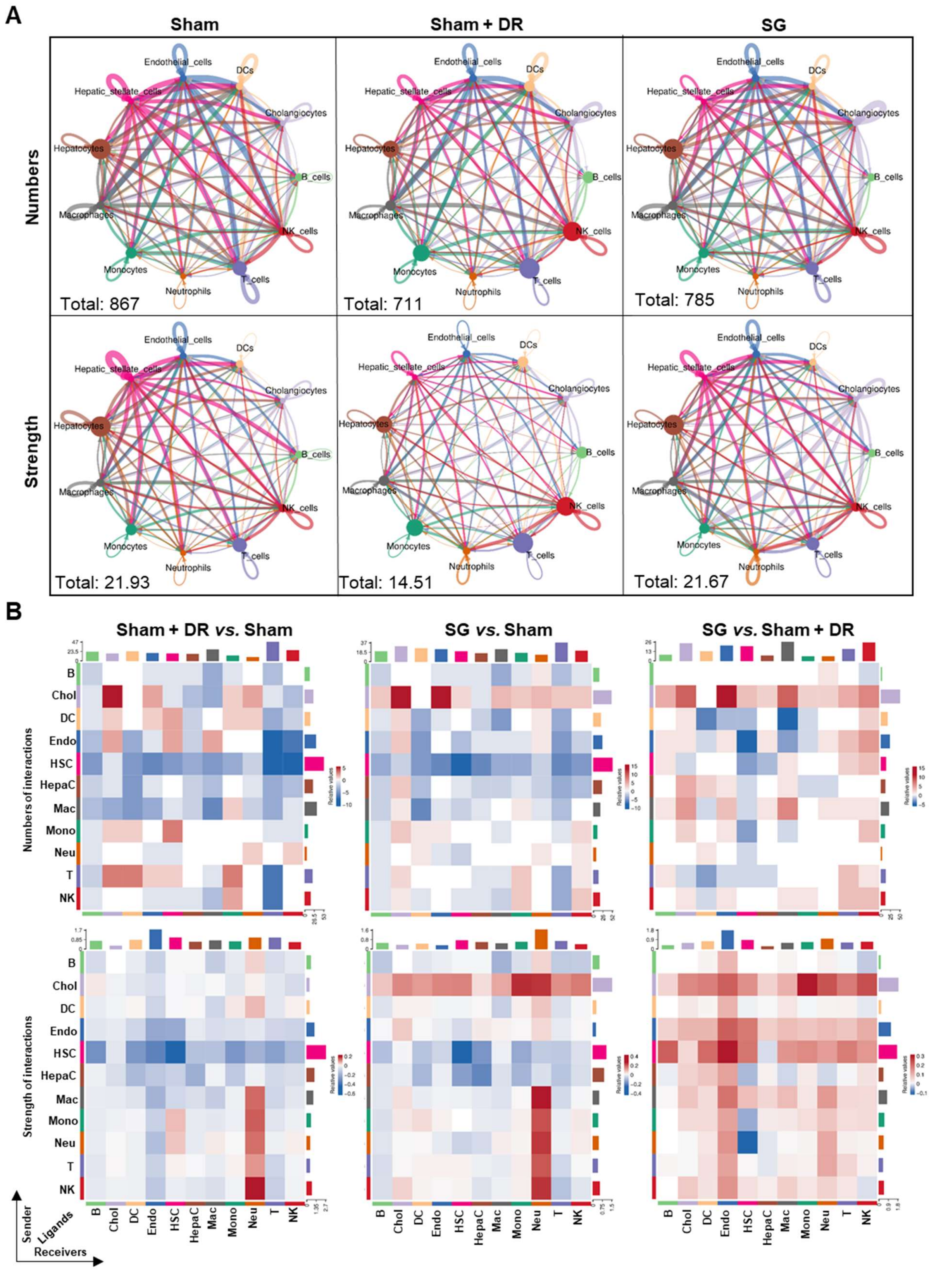

A
Sham
Sham + DR
SG
Numbers
Strength
Endothelial_cells
DCs
Hepatic_stellate_cells
Cholangiocytes
Hepatocytes
B_cells
Macrophages
NK_cells
Monocytes
T_cells
Neutrophils
Total: 867
Total: 711
Total: 785
Total: 21.93
Total: 14.51
Total: 21.67
B
Sham + DR vs. Sham
SG vs. Sham
SG vs. Sham + DR
Numbers of interactions
Strength of interactions
B
Chol
DC
Endo
HSC
HepaC
Mac
Mono
Neu
T
NK
Relative values
Sender
Ligands
Receivers

Fig. S4. Single-cell/single-nuclei RNA-seq analysis deciphers cellular interactions. Numbers and strength of cellular interactions among cholangiocytes, endothelial cells, hepatic stellate cells, hepatocytes, B, T, NK, DCs, macrophages, monocytes and neutrophils in Sham, Sham + DR and SG groups illustrated in **(A**) networks and **(B)** matrixes. Abbreviations: Sham: sham surgery; DR: dietary restriction; SG: sleeve gastrectomy; DC: dendritic cells; NK: natural killer cells; B and B_cells: B lymphocytes; T and T_cells: T lymphocytes; Neu: neutrophils; Mac: macrophages; Mono: monocytes: Endo: endothelial cells; HSC: hepatic stellate cells; Chol: cholangiocytes; HepaC: hepatocytes.

A

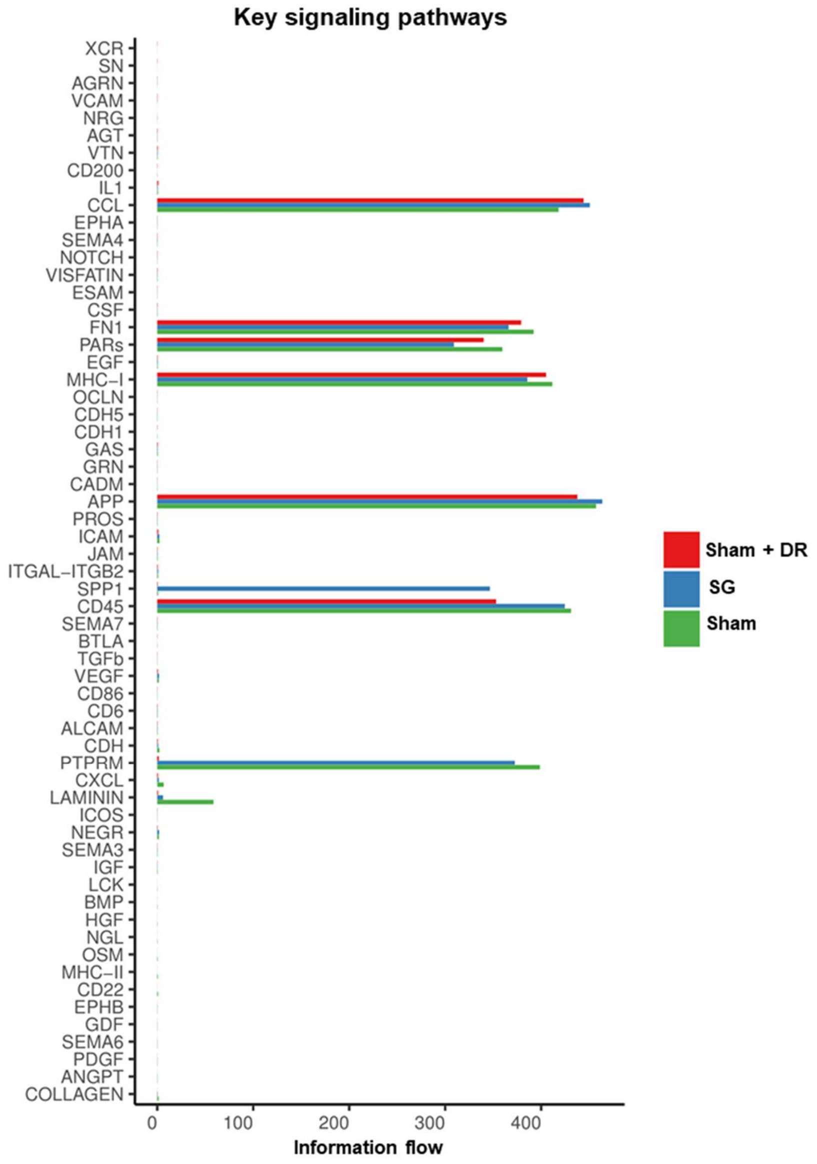

Key signaling pathways
XCR
SN
AGRN
VCAM
NRG
AGT
VTN
CD200
IL1
CCL
EPHA
SEMA4
NOTCH
VISFATIN
ESAM
CSF
FN1
PARs
EGF
MHC−I
OCLN
CDH5
CDH1
GAS
GRN
CADM
APP
PROS
ICAM
JAM
ITGAL−ITGB2
SPP1
CD45
SEMA7
BTLA
TGFb
VEGF
CD86
CD6
ALCAM
CDH
PTPRM
CXCL
LAMININ
ICOS
NEGR
SEMA3
IGF
LCK
BMP
HGF
NGL
OSM
MHC−II
CD22
EPHB
GDF
SEMA6
PDGF
ANGPT
COLLAGEN
0
100
200
300
400
Information flow
Sham + DR
SG
Sham

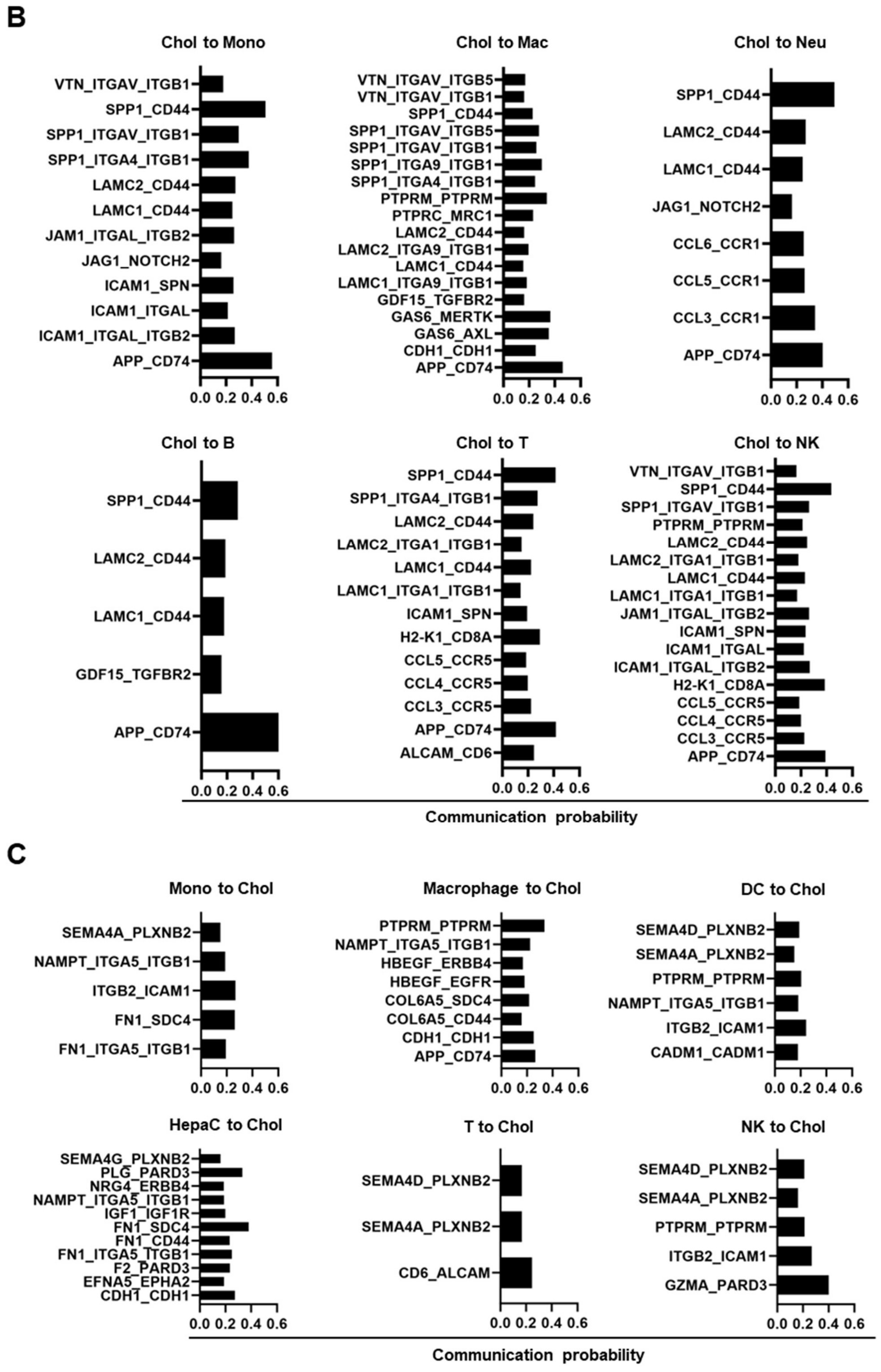

Fig. S5. Single-cell/single-nuclei RNA-seq analysis dissects key pathways in cellular interactions. (A) Frequency of key signaling pathways in Sham, Sham + DR and SG groups.

Significant signaling pathways **(B)** from cholangiocytes to monocytes, macrophages, neutrophils, B, T and NK cells, as well as **(C)** from monocytes, macrophages, DCs, HepaCs, T and NK cells to cholangiocytes. Abbreviations: Sham: sham surgery; DR: dietary restriction; SG: sleeve gastrectomy; DC: dendritic cells; NK: natural killer cells; B: B lymphocytes; T: T lymphocyte; Neu: neutrophils; Mac: macrophages; Mono: monocytes; Chol: cholangiocytes; HepaC: hepatocytes.

A

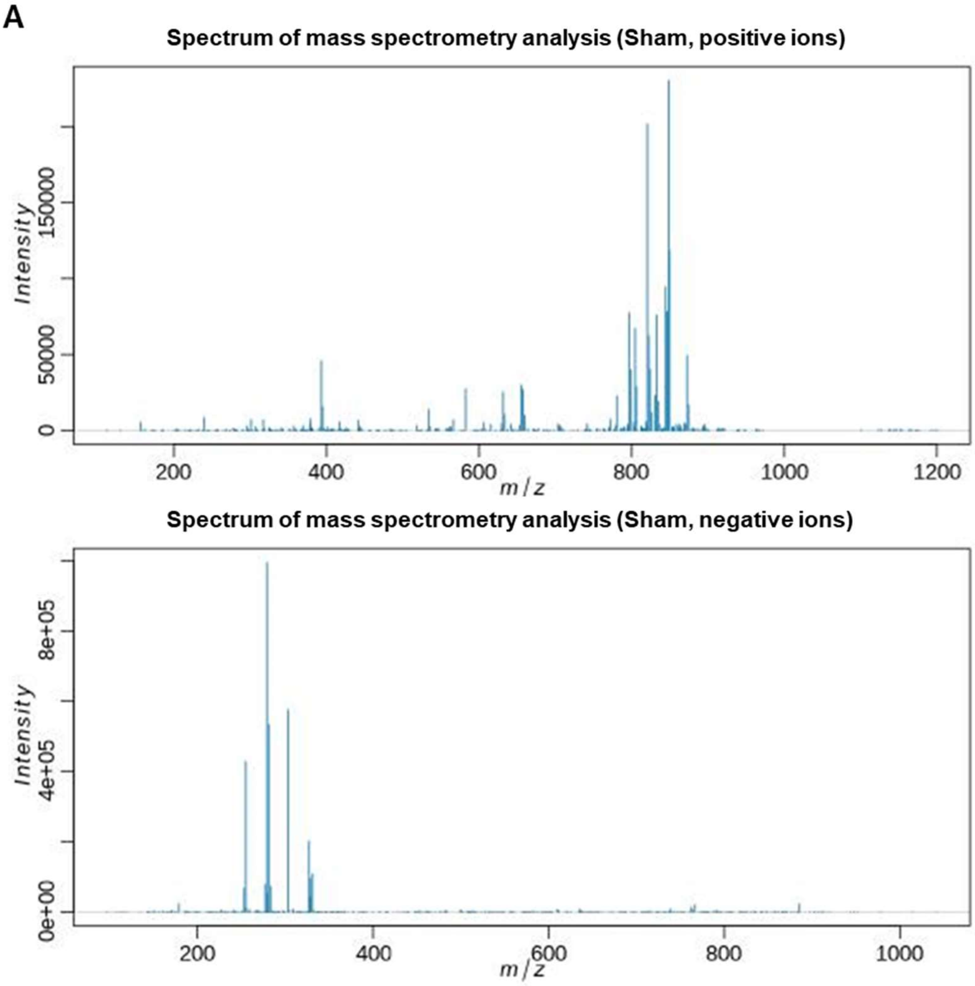
Spectrum of mass spectrometry analysis (Sham, positive ions)
Intensity
0
50000
150000
200
400
600
800
1000
1200
m/z
Spectrum of mass spectrometry analysis (Sham, negative ions)
Intensity
0e+00
4e+05
8e+05
200
400
600
800
1000
m/z

B

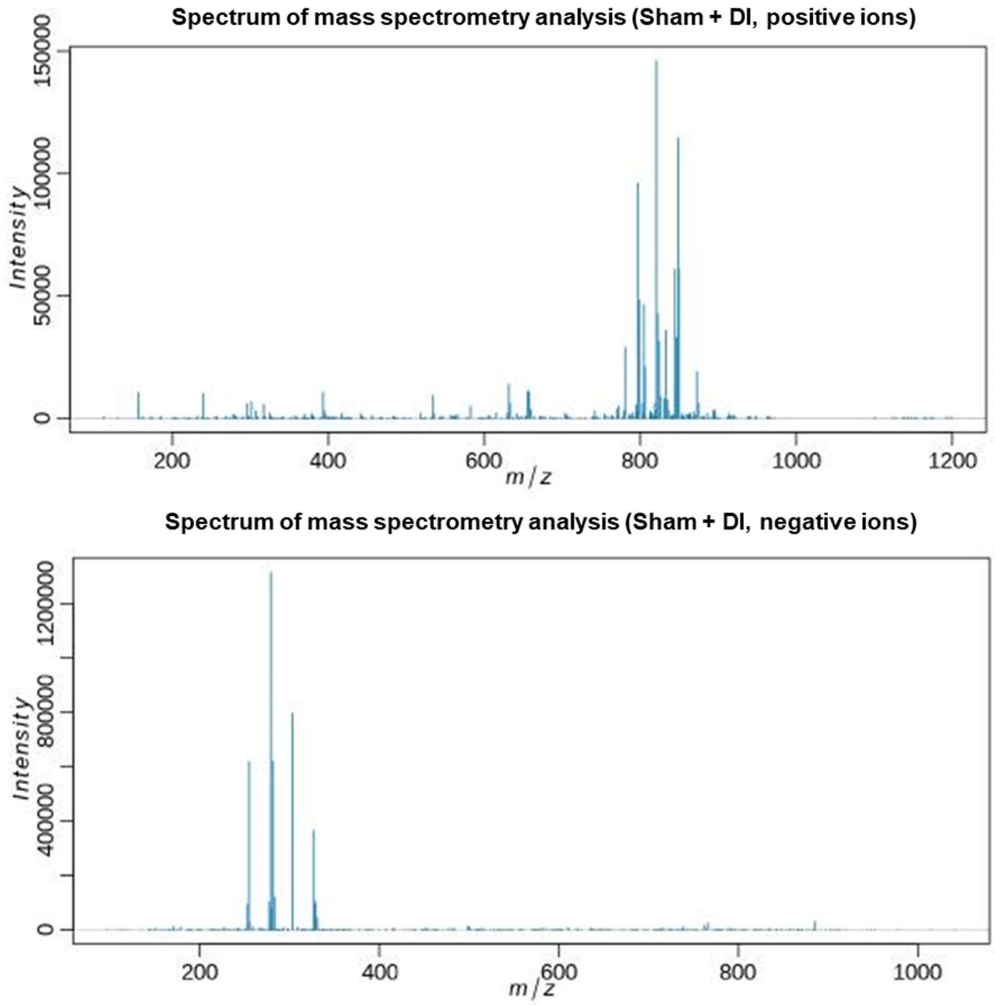
Spectrum of mass spectrometry analysis (Sham + DI, positive ions)
Intensity
0
50000
100000
150000
200
400
600
800
1000
1200
m/z
Spectrum of mass spectrometry analysis (Sham + DI, negative ions)
Intensity
0
400000
800000
1200000
200
400
600
800
1000
m/z

C

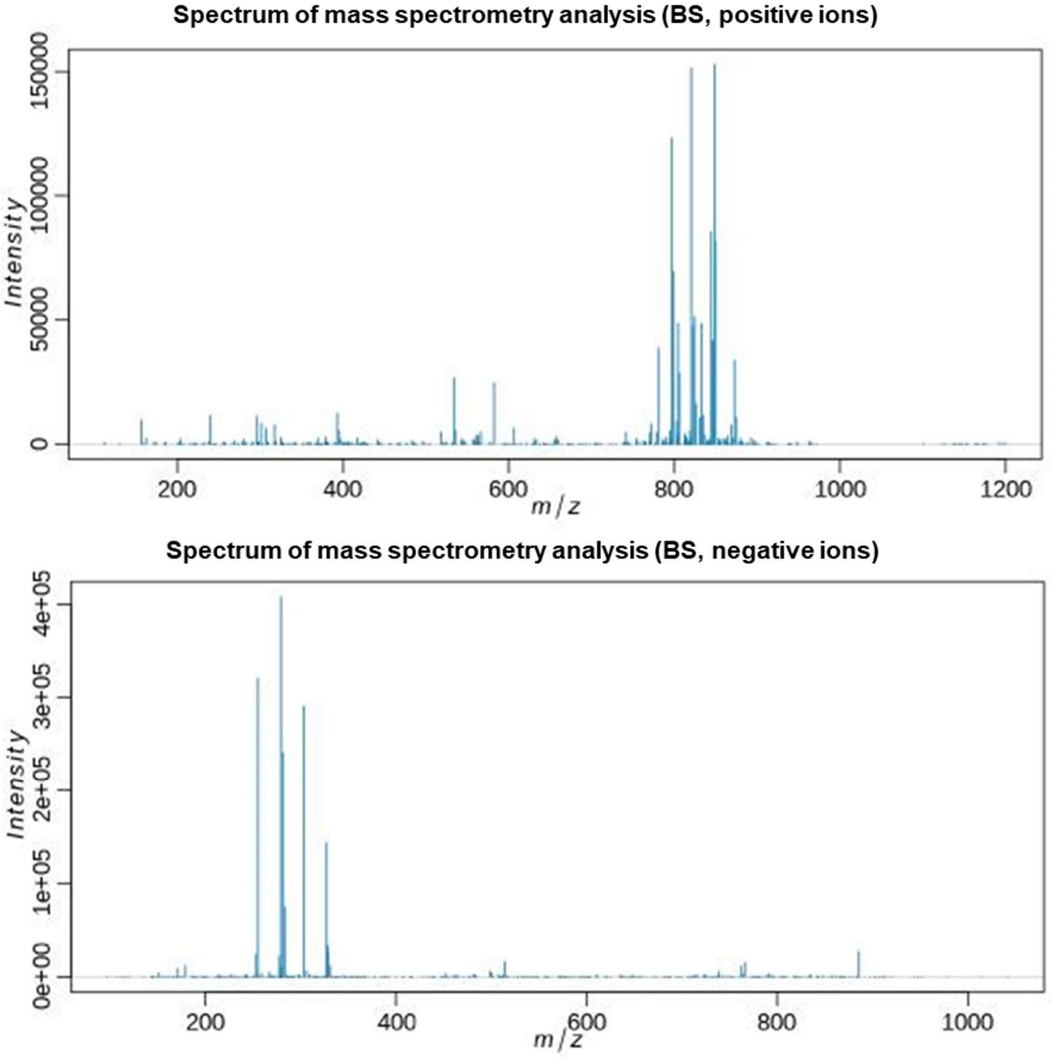
Spectrum of mass spectrometry analysis (BS, positive ions)
Intensity
0
50000
100000
150000
200
400
600
800
1000
1200
m/z
Spectrum of mass spectrometry analysis (BS, negative ions)
Intensity
0e+00
1e+05
2e+05
3e+05
4e+05
200
400
600
800
1000
m/z

D

Metabolite distribution (by positive ions)

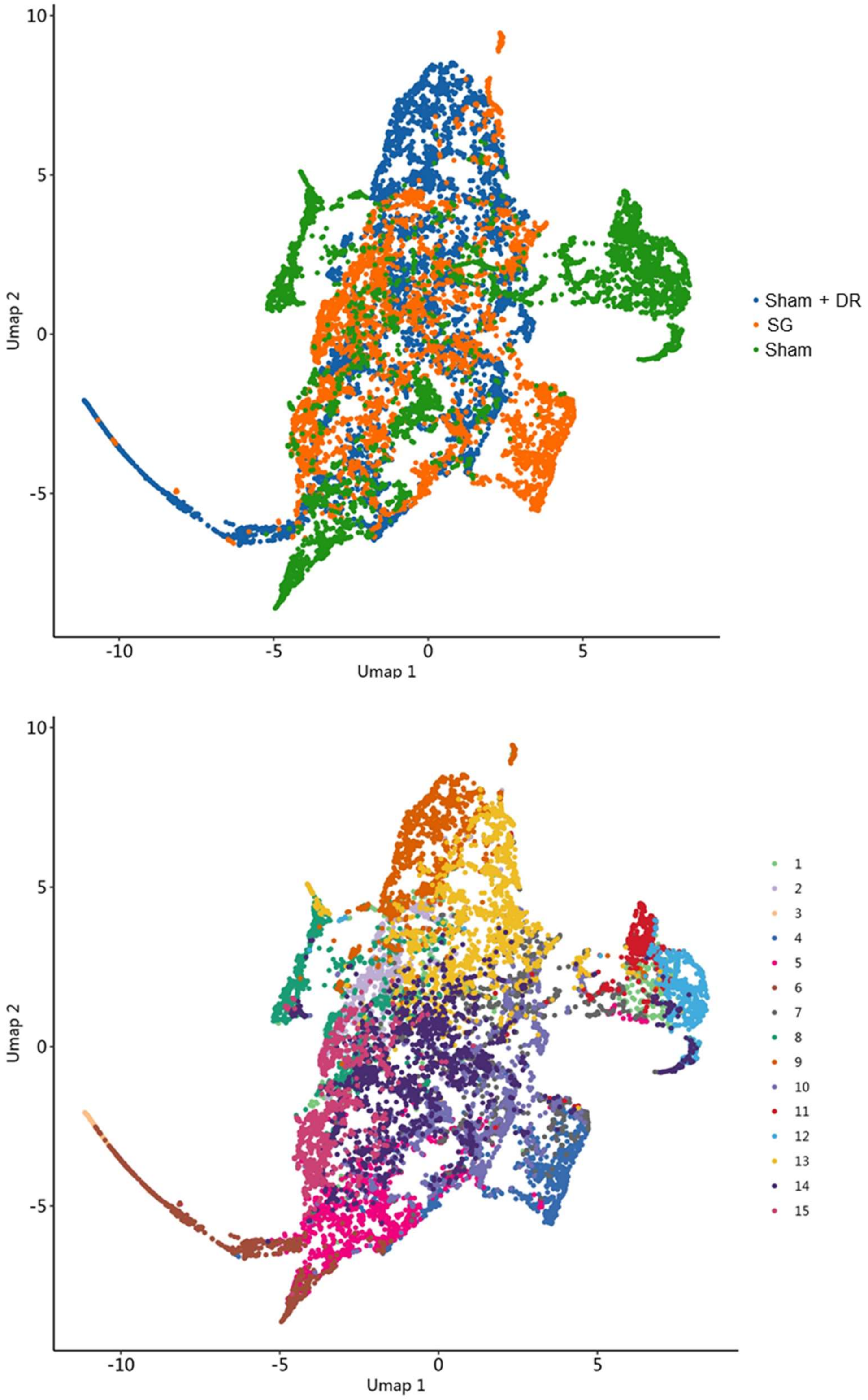

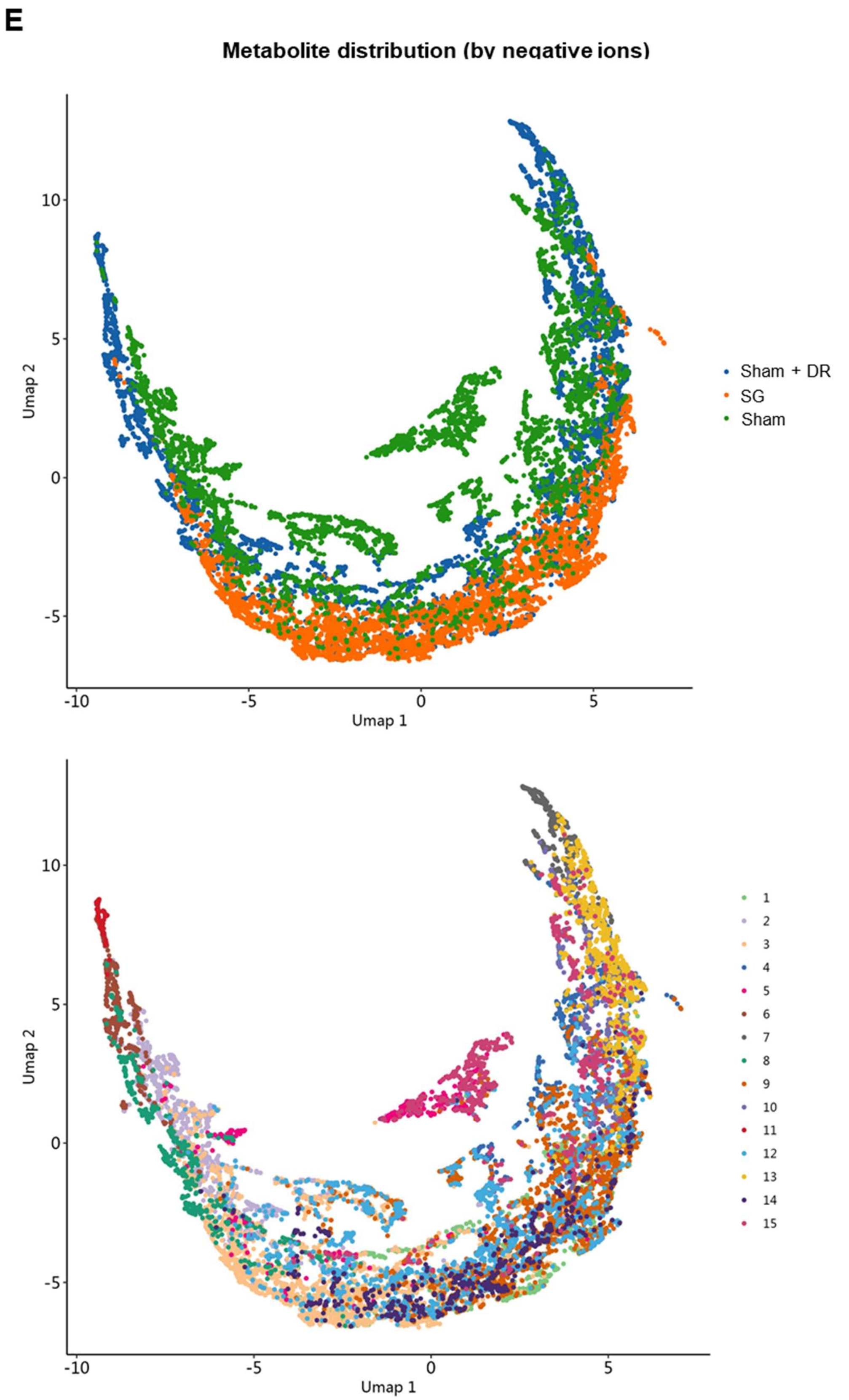
E
Metabolite distribution (by negative ions)
Umap 2
Umap 1
10
5
0
-5
-10
-5
0
5
Sham + DR
SG
Sham
1
2
3
4
5
6
7
8
9
10
11
12
13
14
15

Fig. S6. Metabolomics depicts metabolite profiles and distribution. Spectrum of mass spectrometry detection by positive and negative ions in **(A)** Sham, **(B)** Sham + DR and **(C)** SG groups. The distribution of metabolites detected by **(D)** positive and **(E)** negative ions is illustrated in UMAPs and divided into 15 clusters according to the similarity. Abbreviations: Sham: sham surgery; DR: dietary restriction; SG: sleeve gastrectomy.

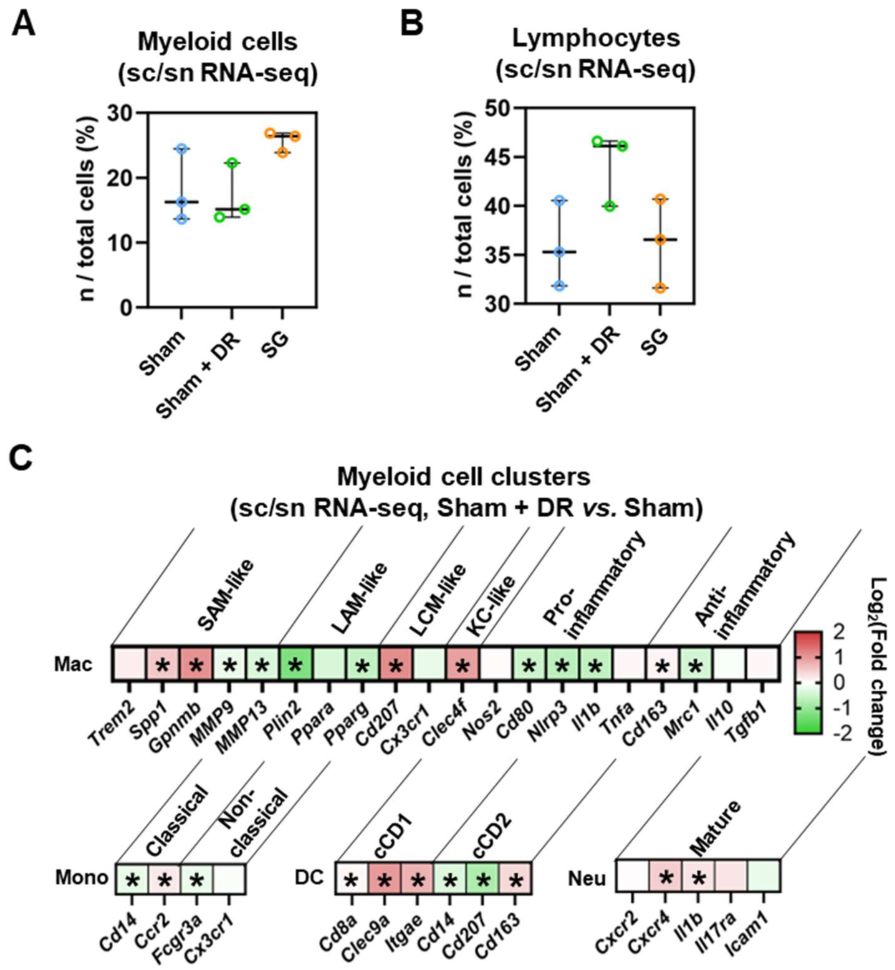

Fig. S7. Assessment of myeloid cells and lymphocytes in rat livers. Proportions of **(A)** myeloid cells and **(B)** lymphocytes per rat liver sample from sc/sn RNA-seq analysis. **(C)** The expression of phenotype-associated markers in myeloid cell clusters was illustrated based on sc/sn RNA-seq data (SG vs. Sham). Abbreviations: C: control; Sham/S: sham surgery; DR: dietary restriction; SG: sleeve gastrectomy; KEGG: Kyoto encyclopedia of genes and genomes. sc/sn: single-cell/single-nuclei; Mono: monocytes; Mac: macrophages; DC: dendritic cells; Neu: neutrophils; SAM: scar-associated macrophage; LAM: lipid-associated macrophage; LCM: liver capsular macrophage; KC: Kupffer cell; cDC: conventional DC. The unpaired t-test and one-way ANOVA test were performed. '*' represents '$p < 0.05$' and statistical significance.

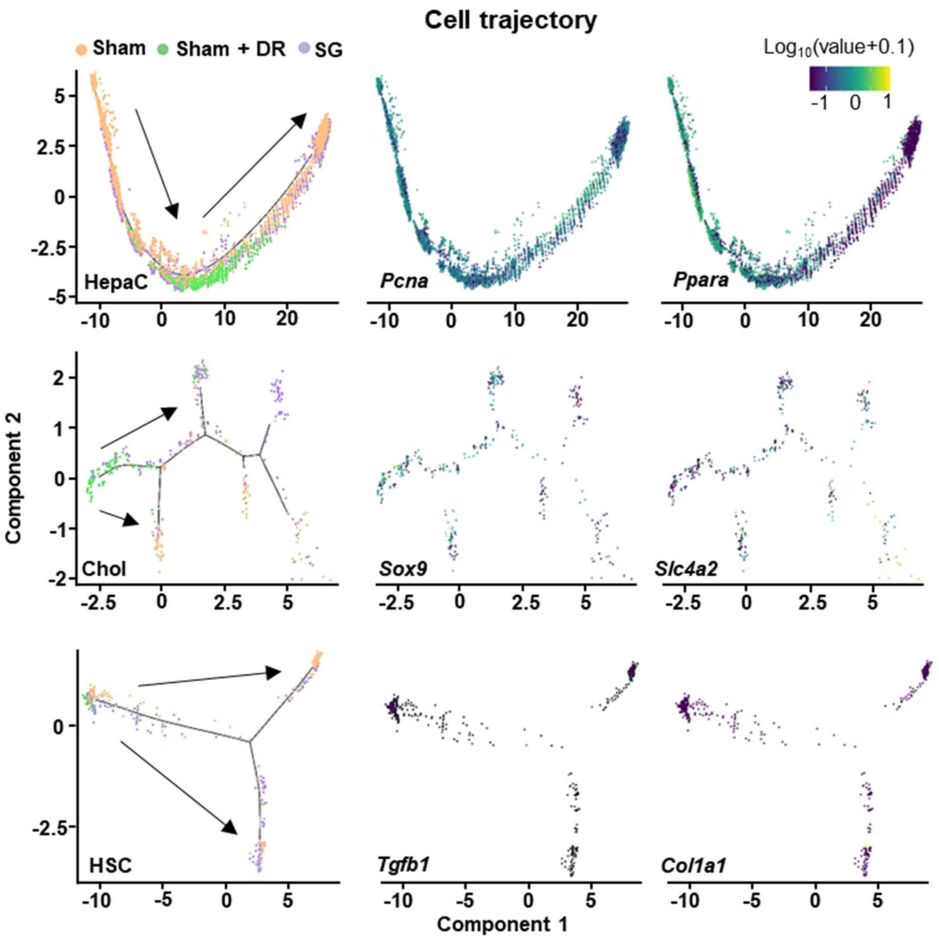

Fig. S8. Single-cell/single-nuclei RNA-seq analysis deciphers cell trajectories. Cell trajectories of HepaC, Chol and HSC, the gene expression of *Pcna* and *Ppara* in HepaC, the gene expression of *Sox9* and *Slc4a2* in Chol, and the gene expression of *Tgfb1* and *Col1a1* in HSC. Abbreviations: Sham: sham surgery; DR: dietary restriction; SG: sleeve gastrectomy; HepaC: hepatocytes; Chol: cholangiocyte; HSC: hepatic stellate cells.

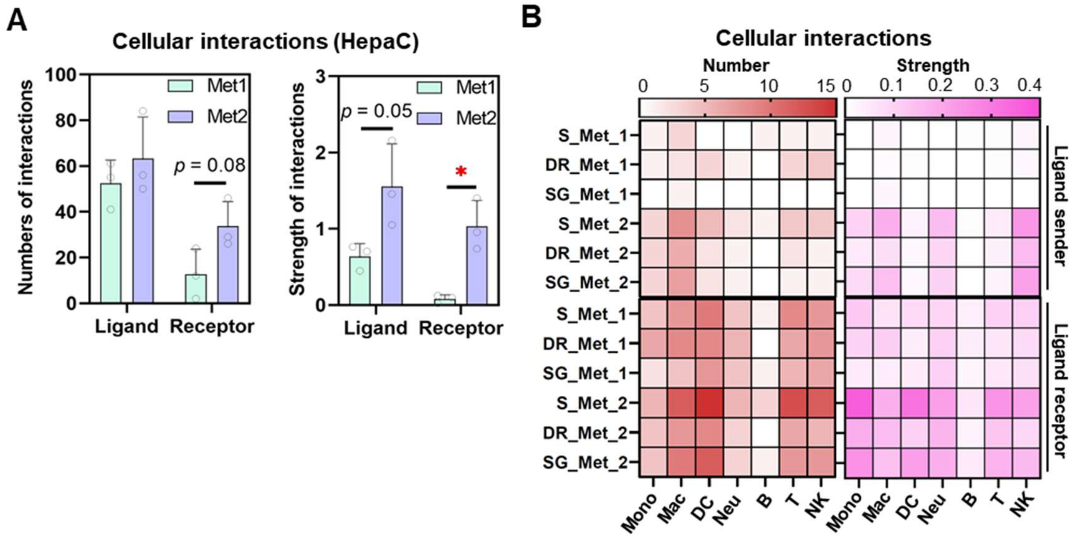

Fig. S9. Cellular interactions in metabolism groups of rat hepatocytes. Numbers and strength of cellular interactions (ligand-receptor) among hepatocytes (Met 1 and 2) and immune cells (B, T, NK, DCs, macrophages, monocytes and neutrophils) displayed in **(A)** total values and depicted in **(B)** matrixes. Abbreviations: Sham: sham surgery; DR: dietary restriction; SG: sleeve gastrectomy; DC: dendritic cells; NK: natural killer cells; B: B lymphocytes; T: T lymphocytes; Neu: neutrophils; Mac: macrophages; Mono: monocytes: HepaC: hepatocytes. The unpaired t-test was performed. '*' represents '$p < 0.05$' and statistical significance.

Table S1. Clinical follow-up data (One day pre-SG vs. six months post-SG)

Total			
**Variables**	**pre-SG (N = 18)**	**post-SG (N = 18)**	***p*-value**
**BMI**	37.01 (34.96 - 39.05)	28.70 (26.81 - 30.58), ↓	<0.05
**PPG** (mmol/L)	9.23 (13.44 - 8.01)	7.23 (9.21 - 7.05), ↓	<0.05
**AST** (IU/L)	38.80 (26.64 - 50.96)	16.56 (13.45 - 19.67), ↓	<0.05
**ALT** (IU/L)	67.28 (38.41 - 96.15)	16.44 (9.03 - 23.85), ↓	<0.05
**γ-GT** (IU/L)	57.89 (34.84 - 80.94)	15.89 (11.49 - 20.28), ↓	<0.05
**TG** (mmol/L)	1.84 (1.25 - 2.44)	1.02 (0.88 - 1.17), ↓	<0.05
**CHO** (mmol/L)	4.82 (4.33 - 5.32)	4.95 (4.49 - 5.41), ↑	0.400
**HDL-C** (mmol/L)	1.19 (1.05 - 1.33)	1.35 (1.21 - 1.48), ↑	<0.05
**LDL-C** (mmol/L)	2.96 (2.49 - 3.43)	3.13 (2.67 - 3.59), ↑	0.419
**TBIL** (μmol/L)	10.67 (8.51 - 12.84)	14.19 (10.20 - 18.18), ↑	<0.05
**ALP** (IU/L)	72.83 (61.04 - 84.63)	74.00 (65.62 - 82.38), ↑	0.849
**TBA** (μmol/L)	2.84 (1.53 - 4.16)	4.88 (0.75 - 10.51), ↑	0.051
**TRF** (g/L)	41.01 (35.21 - 46.81)	36.20 (31.78 - 40.62), ↓	0.175
**IL-6** (pg/mL)	398.18 (316.07 - 480.29)	485.43 (385.89 - 584.96), ↑	0.072
**IL-10** (pg/mL)	33.98 (32.18 - 35.79)	36.55 (34.24 - 38.86), ↑	0.626
**FGF-19** (pg/mL)	491.23 (178.42 - 768.31)	642.16 (255.67 - 880.72), ↑	0.058
**FGF-21** (pg/mL)	1219.67 (1210.03 - 1349.31)	1326.99 (1247.35 - 1419.62), ↑	0.378

Continued

Male			
**Variable**	**pre-SG (N = 8)**	**post-SG (N = 8)**	***p*-value**
**BMI**	39.4 (37.4 - 41.0)	30.8 (29.8 - 32.4), ↓	<0.05
**PPG** (mmol/L)	9.31 (13.44 – 8.24)	7.78 (9.21 – 7.15), ↓	<0.05
**AST** (IU/L)	34 (30 - 63)	16 (14 - 18), ↓	<0.05
**ALT** (IU/L)	55 (48 - 122)	16 (13 - 18), ↓	<0.05
**γ-GT** (IU/L)	82 (35 - 107)	24 (14 - 27), ↓	<0.05
**TG** (mmol/L)	2.01 (1.52 - 3.61)	1.05 (0.93 - 1.21), ↓	<0.05
**CHO** (mmol/L)	4.90 (4.55 - 4.99)	5.32 (4.98 - 5.74), ↑	0.059
**HDL-C** (mmol/L)	1.05 (0.85 - 1.20)	1.22 (1.06 - 1.31), ↑	0.301
**LDL-C** (mmol/L)	2.86 (2.54 - 3.12)	3.71 (3.52 - 4.04), ↑	<0.05
**TBIL** (μmol/L)	12 (9 - 14)	15 (10 - 21), ↑	0.403
**ALP** (IU/L)	74 (58 - 78)	78 (63 - 84), ↑	0.414
**TBA** (μmol/L)	3 (2 - 3)	1 (1 - 3), ↓	0.103
**TRF** (g/L)	40 (36 - 47)	36 (29 - 41), ↓	0.210
**IL-6** (pg/mL)	434 (316 - 456)	423 (342 - 577), ↓	0.703
**IL-10** (pg/mL)	34.2 (32.9 - 35.79)	35.5 (34.24 – 37.79), ↑	0.654
**FGF-19** (pg/mL)	465.43 (178.42 - 723.42)	631.22 (255.67 - 856.23), ↑	0.234
**FGF-21** (pg/mL)	120 (120 - 129)	132 (126 - 136), ↑	0.304

Continued

Female			
**Variable**	**pre-SG (N = 10)**	**post-SG (N = 10)**	***p*-value**
**BMI**	35.1 (32.0 - 36.7)	26.9 (24.0 - 30.2), ↓	<0.05
**PPG** (mmol/L)	9.17 (13.00 – 8.01)	7.18 (8.89 – 7.05), ↓	<0.05
**AST** (IU/L)	28 (17 - 41)	15 (14 - 17), ↓	<0.05
**ALT** (IU/L)	39 (22 - 63)	13 (8 - 15), ↓	<0.05
**γ-GT** (IU/L)	32 (20 - 42)	10 (8 - 10), ↓	<0.05
**TG** (mmol/L)	1.10 (0.95 - 1.45)	0.93 (0.77 - 1.03), ↓	0.121
**CHO** (mmol/L)	4.86 (4.10 - 5.26)	4.70 (3.85 - 5.30), ↓	0.903
**HDL-C** (mmol/L)	1.33 (1.18 - 1.41)	1.41 (1.27 - 1.50), ↓	0.210
**LDL-C** (mmol/L)	2.93 (2.48 - 3.62)	3.09 (2.15 - 3.34), ↑	0.721
**TBIL** (μmol/L)	9.55 (6.90 - 12.40)	11.25 (8.98 - 13.57), ↑	0.304
**ALP** (IU/L)	78 (60 - 93)	77 (66 - 80), ↓	0.700
**TBA** (μmol/L)	1.90 (1.45 - 2.28)	2.25 (1.30 - 2.88), ↑	0.701
**TRF** (g/L)	34 (29 - 49)	32 (30 - 38), ↓	0.913
**IL-6** (pg/mL)	307 (253 - 583)	448 (362 - 552), ↑	0.314
**IL-10** (pg/mL)	33.6 (32.18 - 34.21)	36.7 (35.2 - 38.86), ↑	>0.9
**FGF-19** (pg/mL)	520.13 (213.10 - 768.31)	672.98 (270.81 - 880.72), ↑	0.050
**FGF-21** (pg/mL)	1235 (1149.03 – 1336.03)	1311 (1204.10 – 1509.03), ↑	0.509

Values are presented as 'mean (minimum - maximum)'.
Abbreviation: SG: sleeve gastrectomy; BMI: body mass index; PPG: postprandial blood glucose; AST: aspartate aminotransferase; ALT: alanine aminotransferase; γ-GT: gamma-glutamyltransferase; TG, triglycerides; CHO, cholesterol; HDL-C, high-density lipoprotein cholesterol; LDL-C, low-density lipoprotein cholesterol; TBIL, total bilirubin; ALP, alkaline phosphatase; TBA, total bile acid. TRF: transferrin; IL: interleukin; FGF, fibroblast growth factor. The paired t-test was used. '$p < 0.05$' is considered to be statistically significant.

Table S2. List of antibodies used in this study.

Method	Antigen	Manufacturer	Catalog No.	Clone	Host species	Dilution
IHC	PPAR-α	ThermoFisher	600-401-421	Polyclonal	Rabbit	1/200
	PNPLA3	ThermoFisher	67369-1-IG	1D2B11	Mouse	1/200
	FXR	ThermoFisher	417200	A9033A	Mouse	1/200
	CK7	ThermoFisher	MA1-06315	RCK105	Mouse	1/500
	IL-17A	ThermoFisher	PA5-79470	Polyclonal	Rabbit	1/200
	Ki67	ThermoFisher	MA5-14520	SP6	Rabbit	1/500
	CYP2E1	ThermoFisher	PA5-52652	Polyclonal	Rabbit	1/500
	CYP7A1	ThermoFisher	PA5-100892	Polyclonal	Rabbit	1/200
ELISA	IL-6	ThermoFisher	BS-0782R	Polyclonal	Rabbit	1/1000
	IL-6	ThermoFisher	M620	5IL6	Mouse	1/1000
	IL-10	ThermoFisher	PA5-95561	Polyclonal	Rabbit	1/1000
	TRF	ThermoFisher	A1-46375	57-6	Mouse	1/1000
	FGF-19	ThermoFisher	PA5-79252	Polyclonal	Rabbit	1/1000
	FGF-19	Meibiao biology	MB-7355B	Polyclonal	Rabbit	1/1000
	FGF-21	ThermoFisher	PA5-79255	Polyclonal	Rabbit	1/1000

Abbreviation: PPAR: peroxisome proliferator-activated receptor; PNPLA3: patatin-like phospholipase domain containing protein 3; FXR: farnesoid X receptor; CK7: cytokeratin 7; IL: interleukin; FGF: fibroblast growth factor; CYP: cytochrome P450; TRF: transferrin.
